# A Comprehensive Review on Forward Osmosis Water Treatment: Recent Advances and Prospects of Membranes and Draw Solutes

**DOI:** 10.3390/ijerph19138215

**Published:** 2022-07-05

**Authors:** Yang Xu, Yingying Zhu, Zhen Chen, Jinyuan Zhu, Geng Chen

**Affiliations:** Faculty of Maritime and Transportation, Ningbo University, Ningbo 315211, China; 2011084037@nbu.edu.cn (Y.X.); 2011084024@nbu.edu.cn (Z.C.); 2111084046@nbu.edu.cn (J.Z.); chengeng@nbu.edu.cn (G.C.)

**Keywords:** forward osmosis, draw solute, membrane material, water treatment, water reuse

## Abstract

Forward osmosis (FO) is an evolving membrane separation technology for water treatment and reclamation. However, FO water treatment technology is limited by factors such as concentration polarization, membrane fouling, and reverse solute flux. Therefore, it is of a great importance to prepare an efficient high-density porous membrane and to select an appropriate draw solute to reduce concentration polarization, membrane fouling, and reverse solute flux. This review aims to present a thorough evaluation of the advancement of different draw solutes and membranes with their effects on FO performance. NaCl is still widely used in a large number of studies, and several general draw solutes, such as organic-based and inorganic-based, are selected based on their osmotic pressure and water solubility. The selection criteria for reusable solutes, such as heat-recovered gaseous draw, magnetic field-recovered MNPs, and electrically or thermally-responsive hydrogel are primarily based on their industrial efficiency and energy requirements. CA membranes are resistant to chlorine degradation and are hydrophilic, while TFC/TFN exhibit a high inhibition of bio-adhesion and hydrolysis. AQPs are emerging membranes, due to proteins with complete retention capacity. Moreover, the development of the hybrid system combining FO with other energy or water treatment technologies is crucial to the sustainability of FO.

## 1. Introduction

With the rapid development of the petrochemical industry, ever more wastewater is being produced. The highly saline wastewater produced in petrochemical industrial fields is toxic and difficult-to-degrade industrial wastewater with a salt concentration greater than 1 wt.%, including a certain amount of soluble inorganic salt ions, along with lesser amounts of insoluble impurities and hard-to-remove salts [1,2]. The direct discharge of highly saline wastewater not only causes serious pollution to the environment, but also represents a waste of water resources and salt resources [3,4,5,6]. In addition, global freshwater resources are becoming increasingly scarce [7]. Moreover, the distribution of fresh water is very uneven, and there are still shortages in most areas. These factors have prompted researchers to pay more attention to water recycling technologies and the utilization of water resources.

The existing water treatment methods can be roughly divided into physical, chemical, and biological methods. The physical methods [8] mainly include multi-effect flash evaporation technology, multi-stage flash evaporation technology, and mechanical vapor recompression evaporation, which are energy-intensive and cost-intensive. The chemical methods [9] mainly include incineration, ion exchange, and electrodialysis, which offer high salt-removal efficiencies, but are expensive and often produce harmful by-products. The biological methods [10,11] include the traditional activated sludge method and anaerobic methods, in which the cultivation of functional strains is slow and inefficient, the treated water is difficult to recycle, and secondary pollution is often produced.

**Figure 1:**
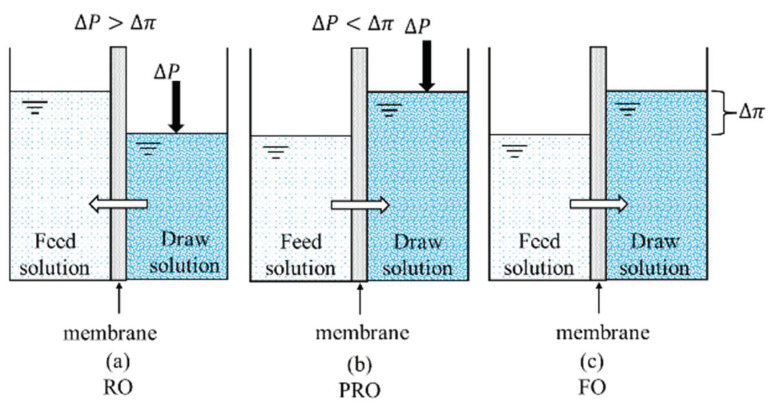
Schematic illustration of RO (**a**), PRO (**b**), and FO (**c**).

**Figure 2:**
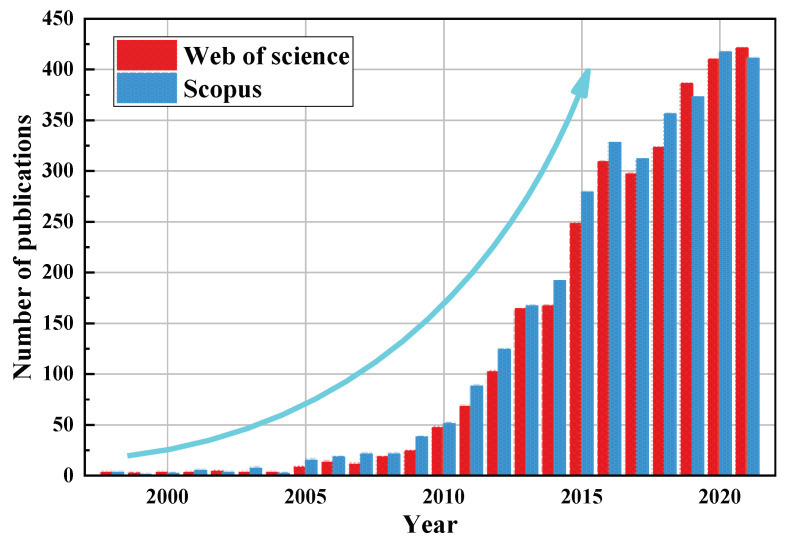
Evolution of publications on FO (last updated on 2 June 2022. The keywords used in searching both Web of Science and Scopus were “forward osmosis”).

Membrane separation, a highly efficient, environmentally friendly, and multi-disciplinary technology, entails various characteristics of the physical, chemical, and biological processes, and has many advantages [12,13,14]. The technology takes advantage of the difference in molecular size of the species in different solutions, uses a polymer membrane as the medium, and relies on concentration differences or external energy as the driving force to separate and purify the required substances [15,16]. It can be divided into different modes, namely reverse osmosis (RO) (Figure 1a), pressure-retarded osmosis (PRO) (Figure 1b), and forward osmosis (FO) (Figure 1c). A schematic illustration of these three membrane separation modes is shown in Figure 1. When the pressure applied on the brine side is greater than the osmotic pressure (Δp > Δπ), the water molecules will be transferred from the high concentration side to the low concentration side. Thus, during operation, a certain amount of external pressure is required as the driving force. When the pressure applied on the brine side is lower than the osmotic pressure (Δp < Δπ), the water molecules will diffuse from the water side to the brine side. The driving force in the FO process is created inherently by the difference in the osmotic pressure between the draw and the feed. This process has many advantages, such as a high recovery rate, a dilute aqueous discharge, relatively low membrane fouling, and no need for external pressure. Hence, FO technology relying on osmotic pressure difference to separate substances is attracting ever more research attention (Figure 2).

In this review, we cover the basic principles and advantages of FO; the major problems affecting the development of FO, including concentration polarization, membrane fouling, and reverse solute flux; and the current advances in terms of FO draw solutes/solutions and FO membrane materials. The future prospects for the sustainability of FO technology in the water treatment process are also discussed.

## 2. Principles and Advantages of FO Water Treatment

### 2.1. Basic Principles of FO Water Treatment

The systems for the FO water treatment are typically composed of three parts: a draw solution; a feed solution; and a membrane showing selective permeability. The membrane lies between the feed solution and the draw solution, separating the two parts. In such a system, the water molecules move from the feed solution side to the draw solution side; the FO process reduces with the decrease in the osmotic pressures and remains relatively static until the osmotic pressures on the respective sides are equal. The diluted draw solution can be reconstituted through secondary separation steps, such as nanofiltration (NF), RO, PRO, distillation, or the application of a thermal, magnetic, or electric field [17,18,19]. The following conditions must be met to realize the operation of the FO process [20]: (1) the draw solute must provide an effective driving force; (2) the selective permeable membrane or membrane module must allow the transport of the water molecules, while preventing the passage of the other solute molecules or ions.

The driving force is the key factor for membrane separation technology. Unlike in RO, the driving force in the FO process is created naturally by the osmotic pressure gradient between the feed solution and the draw solution [20,21]. The osmotic pressure difference determines whether the FO water treatment process can proceed smoothly. In practical applications, the feed solution in the system is often the solution that we need to treat. For example, the highly saline wastewater produced in the petrochemical industrial fields is usually the feed solution in the process of wastewater treatment [6], while seawater is the feed in the process of seawater desalination. The composition of the highly saline wastewater is more complex than that of seawater, as it mainly contains various salts, organic matter, and other substances, which are difficult to treat [22]. The draw solution is a solution system with a higher osmotic pressure, which is composed of a solvent (usually the same as the feed solution solvent; generally water) and a draw solute. For example, sodium chloride with a concentration of 1–2 M was often used as a draw solution in most of the experimental studies. The draw solution is the source of the driving force for the FO water treatment process [23].

The membrane material is the core technology in the FO water treatment process, and its quality directly determines the efficiency of the FO process. An FO membrane is usually composed of a dense active layer for the species’ selection and a porous support layer for the structural strength [24]. The main factors affecting the membrane performance are: concentration polarization; membrane fouling; and reverse solute flux. An ideal FO membrane should have the following characteristics [25]: (i) a dense active layer to ensure the efficient retention of the pollutants; (ii) a thin membrane supporting the layer with high mechanical strength; (iii) good hydrophilicity to achieve a high water flux; and (iv) a good resistance to acids, alkalis, and salts.

### 2.2. Advantages of FO Water Treatment

The FO water treatment offers various prospective benefits when it is deployed on a large scale [26,27]. Firstly, the FO process is driven by an osmotic pressure difference rather than hydraulic pressure, and so consumes less energy, reducing the processing costs. Secondly, compared to traditional pressure-driven membrane processes, such as RO, FO has higher recovery rates, less concentrated aqueous discharges, and is less susceptible to membrane fouling [28]. Furthermore, FO can meet the requirements to treat certain specific effluents, especially some heavy metal wastewaters that are difficult to treat.

**Figure 3:**
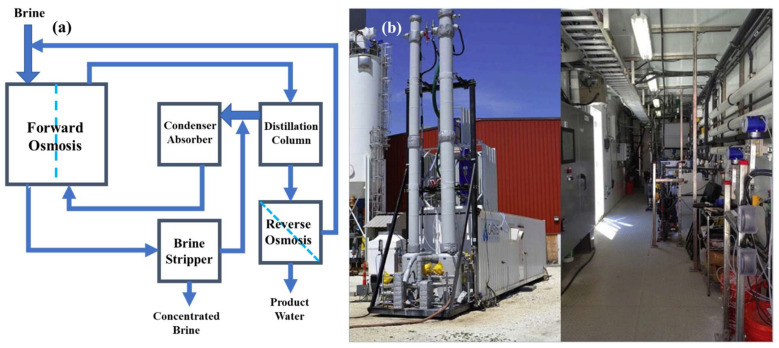
(**a**) Block flow diagram of the membrane brine concentrator (MBC) (darkening color gradation indicates concentration of a stream, while a lightening color gradient indicates dilution; flows from the distillation column and brine stripper to the condenser are gas flows); (**b**) Photographs of the exterior and interior of the FO MBC pilot system [29] (Reprinted with permission from Ref. [29] McGinnis et al., 2013).

In the energy consumption studies, a pilot-scale NH_3_/CO_2_ membrane brine concentrator was tested by McGinnis et al. [29], a block flow diagram of which is shown in Figure 3. The thermal energy consumption of the system was only about 275 kWh per cubic meter of water produced, around 57% lower than that estimated for a traditional concentrator, operated in a similar single-stage, non-mechanical vapor compression configuration. Moreover, for a mechanical vapor compression configuration using electrical energy, the membrane brine concentrator required 42% less electrical energy than a conventional forced-circulation mechanical vapor compression evaporator. A membrane process that is reliant on a thermal drive or hydraulic drive consumes more energy and the process costs can be reduced by using an FO process. Currently, the costs of commercial FO membranes range from tens to hundreds of dollars per square meter, mainly because of low industrial demand. Their cost would be greatly reduced if they were mass-produced [30].

**Figure 4:**
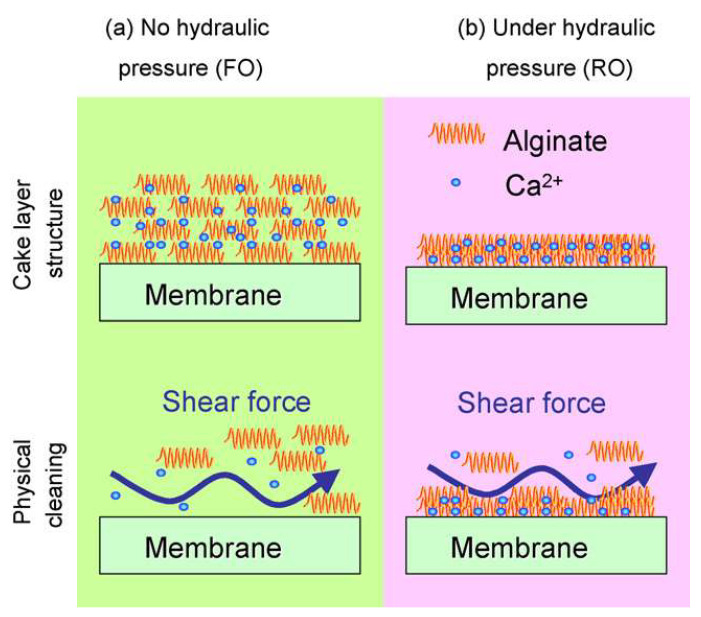
Schematic diagram of fouling and cleaning: (**a**) the lack of hydraulic action causes the alginate fouling layer to loosen, resulting in effective physical cleaning; (**b**) hydraulic action makes the alginate fouling layer dense, resulting in low cleaning efficiency [32] (Reprinted with permission from Ref. [32] Mi et al., 2010).

**Figure 5:**
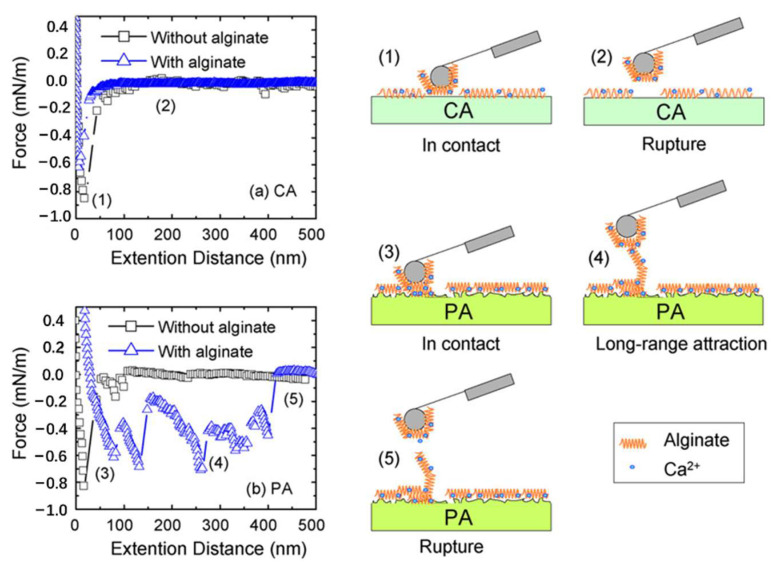
Schematic illustration of cellulose acetate (CA) and polyamide (PA) membranes with/without alginate (all of the test solutions contained 50 mM NaCl and 0.5 mM CaCl_2_): (**a**) representative adhesion force curves for CA membrane with/without 20 mg/L alginate; (**b**) representative adhesion force curves for PA membrane with/without 20 mg/L alginate; (1 and 2) schematic illustration of the retraction of the probe on the surface of the CA membrane; (3–5) schematic illustration of the retraction of the probe on the surface of the PA membrane [32] (Reprinted with permission from Ref. [32] Mi et al., 2010).

The FO is characterized by high recovery rates, dilute aqueous discharges, and good fouling reversibility [28]. To investigate the recovery rates and aqueous discharge concentrations in an FO treatment of wastewater system, Ma et al. [31] conducted a pilot-scale test, in which the desulfurization wastewater was treated with either soda ash or an FO scale inhibitor. Their results showed that the FO system could concentrate such wastewater, with total dissolved solids of 15.6–32.8 g/L in the feed solution to total dissolved solids of more than 120 g/L, that is, a four–eight-fold concentration. More than 99% of the Ca^2+^, Mg^2+^, and Cl^−^ in the wastewater was removed, and the FO system could treat wastewater steadily for a long time. Adding soda ash and FO scale inhibitor, the system recoveries were 85.38% and 73.02%, respectively, which showed the application of FO to the desulfurization wastewater treatment to have higher recovery rates and less concentrated aqueous discharges. To explore membrane fouling in the FO water treatment processes, Mi et al. [32] carried out fouling tests by comparing FO (without hydraulic pressure) and RO (with hydraulic pressure) modes, a schematic diagram of which is shown in Figure 4. Figure 4 shows that only a loose contamination layer structure is often formed in FO mode. The shear force generated by the cross-flow breaks down the loose structure more efficiently in the FO mode than the dense gel layer in the RO mode. The FO achieves a higher cleaning efficiency through simple physical flushing without chemical cleaning. Figure 5 shows the representative adhesion curves obtained for the CA and PA membranes with/without alginate. The maximum adhesion force and breaking distance of these representative force curves are similar to the average values under each corresponding condition. The results showed that the water flux recovery in the former mode was much higher than that in the latter mode, under similar cleaning conditions. The FO proved to be less susceptible to membrane fouling, owing to the less dense organic fouling layer formed without hydraulic pressure, further illustrating that the FO mode may offer unprecedented advantages in reducing or even eliminating the need for chemical cleaning.

**Figure 6:**
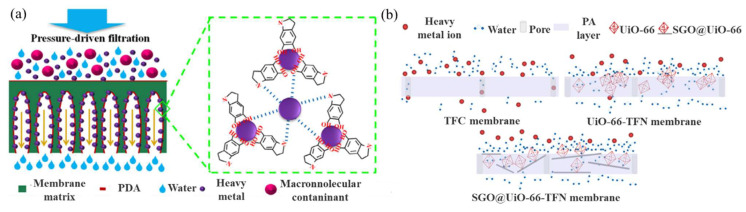
(**a**) Mechanism of the adsorption of heavy metal ions on a PDA membrane [36]; (**b**) heavy metal ion removal and antifouling mechanism of TFC, UiO-66TFN, and SGO/UiO-66TFN [37].

Wastewater containing specific ions can be treated by FO technology. In recent years, the problem of heavy metal pollution in wastewater has attracted widespread concern [33,34]. Certain heavy metals, such as copper and lead, may be deposited in organisms, and have a high ecological impact [35]. Fang et al. [36] designed and fabricated a novel FO membrane by decorating the inner pore walls of a membrane with polydopamine (PDA) nanoparticles for the adsorption of heavy metal ions. Figure 6a shows the possible mechanism of the heavy metal ion removal and antifouling on the PDA membrane. The adsorption properties of the Pb^2+^ on the PES/PDA films were mainly due to the electrostatic attraction between the catechol groups and metal ions, and the N atoms in the NH_2_ groups shared electron pairs to form metal complexes. The results showed that the incorporation of the PDA nanoparticles on the pore walls endowed the synthesized polyethersulfone (PES)/PDA membrane with a significantly enhanced antifouling performance, superior ultrafiltration efficiency, and a high adsorption capacity for heavy metal ions. The static adsorption capacities of several heavy metal ions on the synthesized PES/PDA membrane were 20.23 mg Pb^2+^/g, 17.01 mg Cd^2+^/g, and 10.42 mg Cu^2+^/g, around 1.69, 2.25, and 1.91 times higher, respectively, than those on a conventional PDA-decorated membrane. He et al. [37] modified a thin-film nanocomposite (TFN) membrane with a sulfonated graphene oxide (GO)/metal–organic framework (SGO/UiO-66TFN), to remove heavy metal ions and improve membrane stability. Figure 6b shows the heavy metal ion removal and antifouling mechanism of the thin-film composite (TFC), UiO-66TFN, and SGO/UiO-66TFN. The results showed that the hydrophilic layer of the SGO/UiO-66-TFN membrane could effectively hinder the pollutants and loosen the structure of the pollutant layer, ensuring that the membrane maintained a high removal rate, high water flux (14.77 L/m^2^·h), and high reverse solute flux (2.95 g/m^2^·h). The membrane displayed excellent performance in removing the heavy metal ions, further demonstrating the effectiveness of FO for removing such impurities.

## 3. Major Issues Related to FO

Despite the many favorable characteristics of the FO, there are several challenges that need to be overcome for its application in large-scale processes, including concentration polarization, membrane fouling, and reverse solute flux [38].

### 3.1. Concentration Polarization

All kinds of membrane separation processes, whether pressure-driven or osmotic-driven, generally face the problem of concentration polarization [39]. Theoretically, a large water flux may be achieved by applying a very high osmotic pressure on the side of the FO draw solution, thereby efficiently treating wastewater/seawater. However, the actual flux is often far from the theoretical value, owing to the unique concentration polarization phenomenon in the FO process. The main cause of the concentration polarization is the concentration difference of the solutions on either side of the membrane [40]. Extensive research on concentration polarization has shown that it greatly reduces the effective transmembrane osmotic pressure, and, as such, is one of the major factors contributing to a decline in the water flux and recovery across the semi-permeable membranes [41]. The FO process is subject to both external and internal concentration polarization.

**Figure 7:**
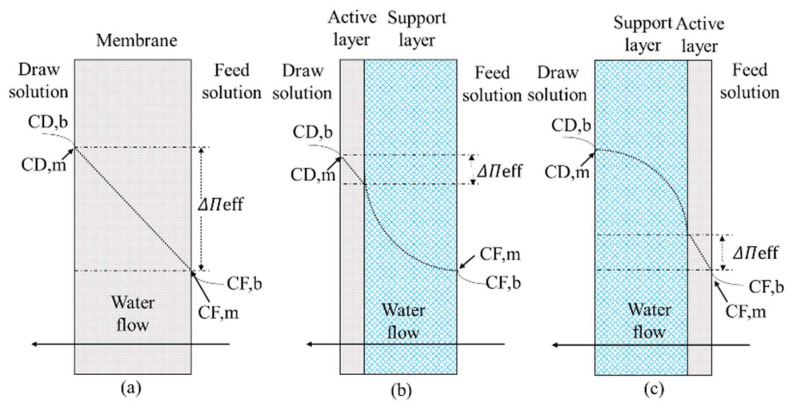
Schematic illustration of concentration polarization in the FO process: (**a**) symmetrical dense membrane; (**b**) asymmetric membrane with the porous supporting layer facing the feed; (**c**) asymmetric membrane with the porous supporting layer facing the draw (C represents the solute concentration; ΔΠeff represents the effective driving force).

The external concentration polarization often occurs with a symmetrical dense membrane, as shown in Figure 7a [42]. When the water on the side of the feed solution is driven to pass through the permeable membrane to the draw side, the solute will be trapped on the surface of the feed side by the dense membrane, thereby inducing concentrative external concentration polarization. Conversely, as the draw side absorbs water from the feed side, the solution in the vicinity of the membrane on the draw side will be greatly diluted, thereby inducing dilutive external concentration polarization. It is evident from the experimental studies that increasing the flow rate, so as to disturb the vicinity of the membrane, can reduce the influence of the external concentration polarization [43].

Internal concentration polarization often occurs in asymmetric membranes [44]. As shown in Figure 7b, when the porous support layer faces the feed side, as the water on the feed side is driven by osmotic pressure through the permeable membrane to the draw side, the solute will accumulate in the porous layer, causing concentrative internal concentration polarization. As shown in Figure 7c, when the porous support layer faces the draw side, as the draw side absorbs water from the feed side, the solution inside the porous layer is greatly diluted, causing the dilutive internal concentration polarization.

It is currently believed that the internal concentration polarization, arising in the porous support layer or fiber support layer, is the main reason why the RO membranes are not suitable for FO processes [45]. Relevant studies [46,47] have indicated that the membrane structure has a great influence on the interaction between the pollutants and the membranes. Therefore, the internal concentration polarization can be effectively reduced by selecting an appropriate membrane material, or changing its structure, or its surface properties. An ideal FO membrane would have no porous support layer or fiber support layer to eliminate the phenomenon of internal concentration polarization, but the strength of such a membrane would be poor [48]. Therefore, it is necessary to optimize the structure of a membrane by balancing the needs for high performance and high strength [49].

### 3.2. Membrane Fouling

Fouling is a dispiriting challenge for many technical and engineering applications. Nearly all of the surfaces drenched in fluid media (e.g., wastewater, seawater, tap water, bodily fluids) are subject to the undesirable settlement and adsorption of foulants/contaminants. Membrane fouling is another factor that affects the performance of FO. Recent studies on membrane fouling in the FO process have led to different conclusions. Mi et al. [32] and Xiao et al. [50] proposed that membrane fouling may not be an important factor affecting the FO process, unlike in RO. Their results showed that the membrane fouling in the FO process is physically reversible, due to the absence of hydraulic pressure, suggesting that chemical cleaning may not be as important in the FO process as in the RO process. However, Tow et al. [51] and Emadzadeh et al. [52] asserted that the problem of membrane fouling has become increasingly prominent, with the expansion of the FO application fields. Similar to other membrane separation technologies, the membrane fouling in the FO process is also affected by various factors, including operating conditions, hydraulic conditions, and the interaction between the pollutants. The contaminants can be deposited on the membrane surface or trapped in the membrane pores or microstructures, so the membrane swing directions (FO mode and PRO mode) have effects on membrane fouling [53].

Phuntsho et al. [54] pointed out that the degree of membrane fouling can be evaluated in terms of membrane filtration resistance calculated, according to Darcy’s law:

(1) $$J=\frac{\Delta P}{\mu (R_{m}+R_{f})}$$

(2) $$J_{0}=\frac{\Delta P}{\mu R_{m}}$$

where *J* is the permeate flux (L/h·m^2^); Δ*P* denotes the transmembrane pressure (Pa); *μ* represents the dynamic viscosity of the solution (Pa·s); and *R_m_* and *R_f_* are the membrane resistance (m^−1^) and the resistance of fouling (m^−1^), respectively.

Membrane fouling is a universal behavior of FO membranes and is closely related to surface hydrophilicity, morphology, charge, and other properties. Wang et al. [55] explored a dual cleaning approach, to block inorganic fouling and biofouling. In their approach, ethylenediaminetetraacetic acid (EDTA) was first added to the draw solution to minimize inorganic fouling. However, this favored biofouling, as the EDTA constituted an extra nutrient for bacterial proliferation. To remedy this, the second part of their strategy was to chemically clean the membrane support layer with NaClO solution to control biofouling. The membrane surface modification is one of the most common and versatile methods for improving the separation and antifouling performances of TFC [56] and cellulose triacetate (CTA) membranes [57]. The mechanism of membrane fouling is related to electrostatic repulsion. Choi et al. [58] synthesized functionalized multi-walled carbon nanotube-blended cellulose acetate (fCNT-CA) membranes and investigated the alginate-fouling mechanism thereon. Their results revealed that the fCNTs enhanced the alginate-fouling resistance in FO (a 57% lower normalized water flux decrease was incurred for a 1% fCNT-CA membrane compared with the bare CA membrane), which was attributed to enhanced electrostatic repulsion between the membrane and the alginate contaminant. However, the bio-identification of this membrane fouling treatment method is still insufficient. Luo et al. [59] complemented the biological treatment by utilizing an aquaporin (AQP) FO membrane, and examined its performance in removing 30 trace organic contaminants (TrOCs). Their results showed that all of the 30 selected TrOCs were more than 85% removed, regardless of their diverse properties. Thus, the AQP membrane complemented the biological treatment for stable and excellent foulant removal.

### 3.3. Reverse Solute Flux

In the FO process, reverse solute flux from the draw solution to the feed solution seems to be an unavoidable problem, due to the concentration gradient. Such reverse diffusion from the draw solution will seriously affect the FO process. In this way, sewage can be contaminated by multivalent ions in the feed solution, which will aggravate the FO membrane fouling. Meanwhile, the internal concentration polarization may be increased, due to the larger ion size and lower solution diffusion coefficient [60]. Many studies have shown that the reverse solute flux, in the same way as the water flux and salt rejection rate, has become an important parameter to measure FO performance. Choi et al. [58] evaluated water-permeation flux and reverse solute flux using a synthesized fCNT-CA membrane and a commercial FO membrane from HTI Co., whereby the reverse solute flux was calculated as follows:

(3) $$J_{s}=\frac{c_{F}V_{F}}{A_{m}t}$$

where *J_S_* is the calculated reverse solute flux; *c_F_* denotes the final NaCl concentration in the feed after operation; *V_F_* represents the final volume of the feed solution after operation; and *A_m_* and *t* are the effective membrane area and time.

Yong et al. [61] investigated the reverse fluxes of three neutral draw solutes—urea, ethylene glycol, and glucose—across an asymmetric FO membrane. The experimentally measured water fluxes were consistently lower than the theoretically calculated water fluxes during the osmotic-driven membrane processes, even taking into account the effects of the external concentration polarization, demonstrating the effect of the solute–solvent coupling on reverse flux selectivity (i.e., the ratio of forward water flux to reverse solute flux). In order to better represent the membrane performance, the specific reverse solute flux (SRSF, i.e., the ratio of the reverse solute flux to the water flux) was introduced as a standard parameter to measure [62]. A larger ratio represents worse FO performance.

The above restrictions on FO development, namely the concentration polarization, membrane fouling, and reverse solute flux, are controlled by the factors such as membrane orientation, inadequate membrane structure, the concentrations of the draw and feed solutions, and the operating conditions [63]. Recently, many studies have been based on the dual aims of improving draw solution performance and devising excellent membrane materials to reduce the impact of the above limitations; thereby, enhancing the efficiency of the FO process.

## 4. Advances in FO Draw Solutes/Solutions

An ideal FO draw solute/solution often needs to satisfy the following three requirements [64]: high water-permeation flux; easy recovery with low energy requirements; and low reverse solute flux. The draw solutes can generally be classified into five types, namely: gaseous draw solutes; inorganic-based draw solutes; organic-based draw solutes; magnetic nanoparticles (MNPs); and hydrogels. In most FO water treatment, sodium chloride was commonly used. Table 1 shows the different draw solutes/solutions used in the studies of the FO water treatment.

### 4.1. Gaseous Draw Solutes

**Figure 8:**
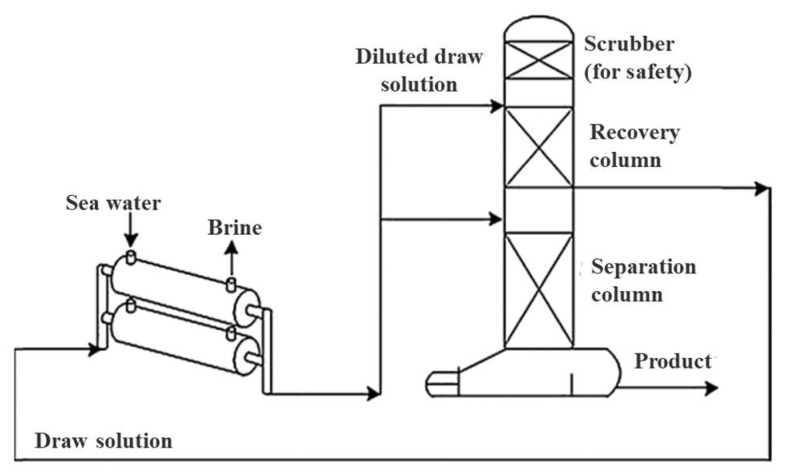
FO desalination system with ammonium NH_4_HCO_3_ as the draw solute [65] (Reprinted with permission from Ref. [65] Kim et al., 2015).

Certain acidic or alkaline gases, such as CO_2_, SO_2_, and NH_3_, showing good solubility in water, can provide a large osmotic pressure and have been widely used as draw solutes in the FO processes. Moreover, these gases can be easily separated and recovered from the water through heating, which is conducive to the realization of recycling in the FO process. Kim et al. [65] used ammonium hydrogen carbonate (NH_4_HCO_3_) as a draw solute to extract water from highly saline feed solution. As shown in Figure 8, the large osmotic pressure generated by the highly soluble NH_4_HCO_3_ produced a high permeation flux of 6.8 L/m^2^ h product water, which in turn led to a high water recovery rate of 99.9%. It was concluded that FO desalination with NH_4_HCO_3_ solution could produce water of high purity. However, the results showed that the thermal energy consumption of the system was 265–300 kWh/m^3^ of produced water, which is higher than the conventional MSF desalination process (38 kWh/m^3^ thermal energy and 3.5 kWh/m^3^ electrical energy). Therefore, the gaseous solutes are seldom used in real applications, due to their limited osmotic pressure and the energy required for their separation and recovery.

### 4.2. Inorganic-Based Draw Solutes

**Figure 9:**
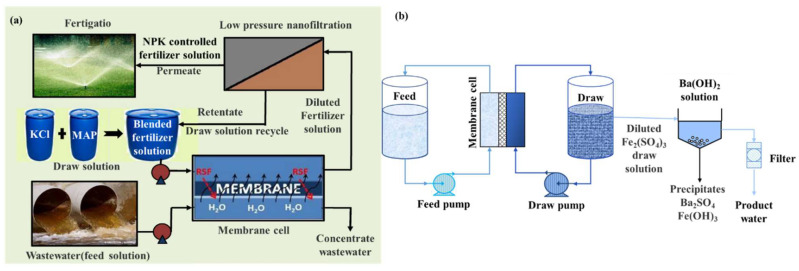
Schematic diagram of several inorganic-based draw solutes: (**a**) concept of fertilizer-drawn FO process [66] (Reprinted with permission from Ref. [66] Dutta at al., 2019); (**b**) schematic diagram of FO desalination process of iron(III) sulfate extraction solution [64].

Inorganic salts, such as NaCl, MgCl_2_, KNO_3_, and MgSO_4_, can also be used as draw solutes in FO processes, by virtue of their good water solubilities and high osmotic pressures. To demonstrate the effectiveness of an integrated fertilizer-driven FO system, Dutta et al. [66] introduced an application of the dehydration of wastewater and brackish water, using inorganic fertilizers (KCl and NH_4_PO_3_) as draw solutes in a bench-scale unit with an ultra-low-pressure membrane, as shown in Figure 9a. An advantage of this application is that the diluted fertilizer draw solution can be directly used for fertilization, instead of separating the draw solute from the desalinated water. The results showed that reversible fouling was observed with >90% flux restoration, and low reverse solute flux of 1.74 g/m^2^ h NH_4_H_2_PO_4_ was achieved for the dewatering of the brackish water and wastewater concentration. However, the water flux of more than 2.72 L/m^2^ h was moderate. In terms of energy consumption, the energy consumption per cubic meter of diluted fertilizer solution was 8.8 kWh. Phuntsho et al. [54] also used different fertilizers (KCl, NaNO_3_, NH_4_Cl, and (NH_4_)_2_SO_4_) as the draw solutes for saline water desalination. According to the preliminary calculations, 1 kg of fertilizer could draw 11–29 L of water from a brackish water source. The KCl showed the highest pure water flux (22.85 L/m^2^ h), followed by NaNO_3_, NH_4_Cl, and (NH_4_)_2_SO_4_. The reverse solute flux of KCl (59.6 g/m^2^ h) was also the smallest. Similarly, but more economically, Volpin et al. [67] used a commercial fertilizer blend to concentrate real diluted urine. The experiments, using 2 L of real urine as the feed and 0.5 L of 0.5 M Mg(NO_3_)_2_·6H_2_O as the draw, were performed. During the concentration process, the urea in the urine was recovered as it diffused into the fertilizer. With 50% concentrated urine, 93% of the P was recovered, without the addition of external Mg^2+^; 50% N recovery was achieved in the diluted fertilizer draw solution. An economic analysis indicated that the total revenue of the process would be over 5.3 times the associated costs.

In addition to being used in fertilizers, inorganic solutes can also be separated from fresh draw solutions by precipitation. As shown in Figure 9b, Qasim et al. [64] studied the feasibility of using iron(III) sulfate as a draw solute in the FO process, and used a barium hydroxide precipitation method to obtain fresh water from the diluted draw solution used to treat the raw material liquid. They desalinated synthetic brackish water (5000 ppm NaCl) and seawater (40,000 ppm NaCl) using a commercial FO membrane (cellulose triacetate-based) with a 280,000 ppm ferric sulfate as the draw solution under ambient conditions. The results presented that the measured mean water fluxes were 3.75 and 1.61 L/m^2^ h for the treatment of the brackish water and seawater, respectively. Using deionized water as the feed solution, a reverse ferric sulfate flux of 1.88 g/m^2^·h was observed, which showed that the recycling of reclaimed water has obvious benefits. This method simultaneously meets the requirements of treatment of the feed solution and the recycling of fresh water, and may be widely used in the areas where water resources are scarce.

### 4.3. Organic-Based Draw Solutes

**Figure 10:**
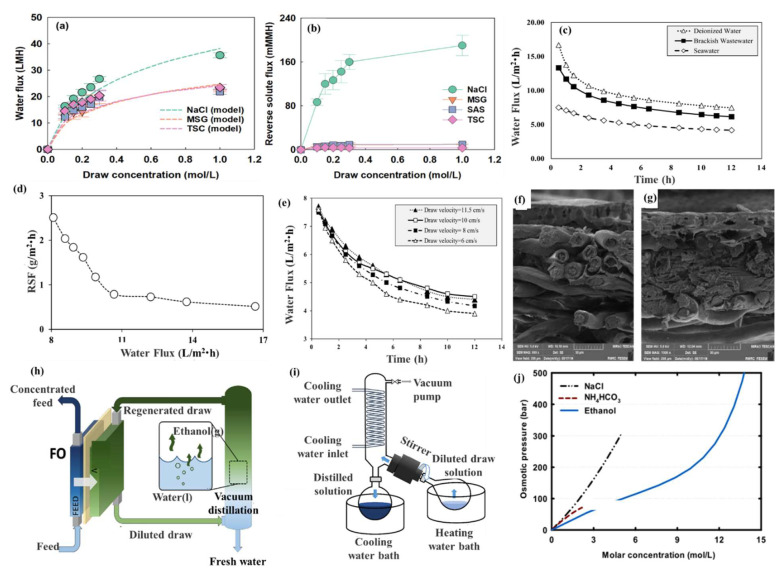
(**a**) water flux (points: experimental data, line: modeled data) with increasing draw concentration (SAS was not modeled because its diffusion coefficient could not be obtained from the literature); (**b**) comparison of reverse solute fluxes using food additives [68] (Reprinted with permission from Ref. [68] Yang et al., 2021); (**c**) variation of water flux with operation time at a controlled flow rate of 10 cm/s; (**d**) variation of reverse solute fluxes with water flux for deionized water feed and molasses draw; (**e**) effect of molasses draw flow velocity on water flux at a seawater feed velocity of 10 cm/s; (**f**) cross-sectional FESEM image of a pristine membrane in the molasses-drawn FO system; (**g**) cross-sectional FESEM image of a fouled membrane [69] (Reprinted with permission from Ref. [69] Bagheri et al., 2021); (**h**) schematic diagram of ethanol as a draw solute for FO; (**i**) schematic diagram of a laboratory-scale batch vacuum distillation unit; (**j**) experimentally measured water and reverse solute fluxes for NaCl, NH_4_HCO_3_, and ethanol draw solutions [70] (Reprinted with permission from Ref. [70] Kim et al., 2019).

Compared with inorganics, organics tend to have larger molecular masses, so using them for solute reverse osmosis is not obvious. The commonly used organic extracting draw solutes are based on sugars, diethyl ether, and organic salts. Yang et al. [68] selected three commonly used food additives (MSG, SAS, and TSC) as draw solutes. The osmotic pressures provided by these food additives were slightly higher than that with NaCl. As shown in Figure 10a, the FO water-permeation fluxes of these additives were up to about 20 L/m^2^·h, 70–78% lower than that with NaCl, mainly due to a severe internal concentration polarization. However, reverse solute fluxes with these additives were only 3–9% of that with NaCl, indicating less contamination (Figure 10b). Molasses, a common and typical organic substance, exhibits a poorer water flux performance as a draw than the first three additives. Bagheri et al. [69] obtained fluxes of 16.7, 13.3, and 7.5 L/m^2^·h using molasses as the draw over a period of 30 min against feeds of deionized water, brackish wastewater, and seawater, respectively (Figure 10c). The reverse solute flux was increased by decreasing the water flux (Figure 10d) and was more significant at deionized water feeds lower than 10.67 L/m^2^·h. In addition, Figure 10e shows an increase in the water flux from 4.1 to 4.4 L/m^2^·h with increasing feed cross-flow velocity (from 6 to 11.5 cm/s) after 12 h. The reason for the improvement in the water flux by increasing the draw velocity was the decrease in the effect of the external concentration polarization. It can be seen from Figure 10f,g that the membrane has an asymmetric structure, including a dense top layer and a porous support layer. The fouling material has entered slightly into the support layer and deposited on the backside of the active layer, filling the voids between the fibrils, as well as the pores to some extent. Ethanol is an organic substance, but its principle is similar to that of gaseous draw solutes. Kim et al. [70] evaluated ethanol for its potential as a draw solute in FO in terms of the performance in treating highly saline wastewater, and the energy requirements for regeneration (Figure 10h,i). Compared to NH_4_HCO_3_, as shown in Figure 10j, the ethanol exhibited a higher osmotic pressure and easier regeneration. In addition, ethanol, as an organic-based draw solute, is rarely used because of its high cost, but it still has certain potential due to its high solubility and high osmotic pressure. Last but not least, the recycling of organic-based draw solutes requires an energy-intensive process, such as ultrafiltration or membrane distillation, which requires careful scrutiny. Zhao et al. [71] applied polyacrylamide as a draw solute in an FO process. A dilute polyacrylamide solution maintained high viscosity and could be directly used for polymer flooding in many of the oil fields to increase oil production. Last but not least, the recycling of organic-based draw solutes requires an energy-intensive process, such as ultrafiltration or membrane distillation, which requires careful scrutiny.

### 4.4. MNP-Based Draw Solutes

**Figure 11:**
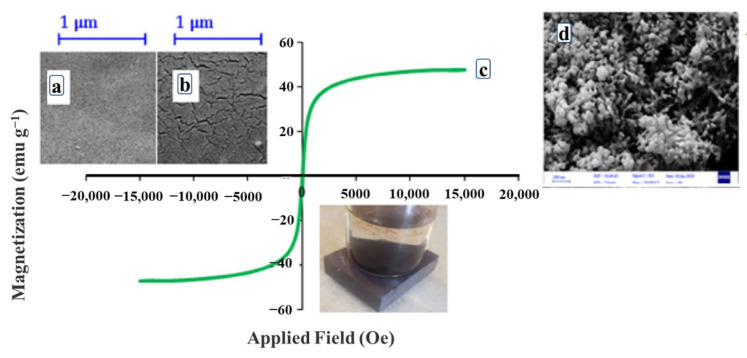
SEM images of fresh CTA membrane (**a**) and used membrane (**b**); magnetization curves (**c**) of Fe_3_O_4_@SiO_2_-SA in recovery process; and (**d**) FESEM images of Fe_3_O_4_@SiO_2_-SA after recovery [73] (Reprinted with permission from Ref. [73] Khazaie et al., 2021).

A large amount of energy is often consumed in the process of recovering the draw solutes [76]. To circumvent this, magnetic draw solutes, due to their easy separation and low reverse solute flux, have become a hot spot in current research [77]. The MNPs are composed of a magnetic core enwrapped by hydrophilic organic ligands [78]. The MNPs are well-known filler materials that can be attached to polymers to impart hydrophilicity, magnetic properties, and, hence, easy recyclability. Kim et al. [79] investigated the possibility of magnetic separation in desalination using magnetic nanoparticles. The experimental study and numerical simulation analysis show that the amount of magnetic nanoparticles captured in the separation column increases with the increase in the magnetic field strength and particle size. As a result, in order to realize the separation and reuse of draw solutes by magnetic separation, it is necessary to develop a new type of nanoparticle magnetic separator and a large-scale high-performance magnetic separator at the same time. Shoorangiz et al. [72] synthesized d-xylose-coated MNPs for use as a draw agent in an FO process for water purification. Using deionized water as the feed, the initial FO water flux was 2.98 L/m^2^·h. Reusing the recovered MNP draw agent in two further consecutive tests resulted in reductions in the water flux to 2.68 and 2.30 L/m^2^·h, respectively, demonstrating good reusability. Khazaie et al. [73] modified the surface of the Fe_3_O_4_ nanoparticles with sodium alginate (SA) to enhance their dispersibility and stability in the aqueous solution. Figure 11c demonstrates how quickly and straightforwardly the coated magnetic draw solute can be collected from the solution and clear water is perceived. Its performance is further improved compared to d-xylose-coated MNPs, the average water flux of 0.06 g/mL Fe_3_O_4_@SiO_2_-SA in FO mode is 8.5 L/m^2^·h, and the reverse solute flux is 0.23 g/m^2^·h. The high stability and simple recovery of the draw solute decreases the cost of desalination, verifying its reusability.

In general, the main advantage of the MNP-based draw solutes lies in their extremely high surface area to volume ratio [80]. Compared with the inorganic-based and organic-based draw solutes, the MNPs have larger molecular sizes, which facilitates their recovery from the low-pressure membrane processes with the aid of a magnetic field or by micro-/nanofiltration. Thus, they offer high stability, simple regeneration, and reduced water production costs [81]. Moreover, although the MNPs are non-electrolytes, they can generate very high osmotic pressures (up to 70 atm for the MNPs coated with polyacrylic acid), much higher than that of seawater (26 atm), which makes the osmotic process very effective [82]. Nevertheless, conglomerations of the nanoparticles may remain in the water, which limits the practical application of the hydrophilic superparamagnetic nanoparticles [74].

### 4.5. Hydrogel Solutes

**Figure 12:**
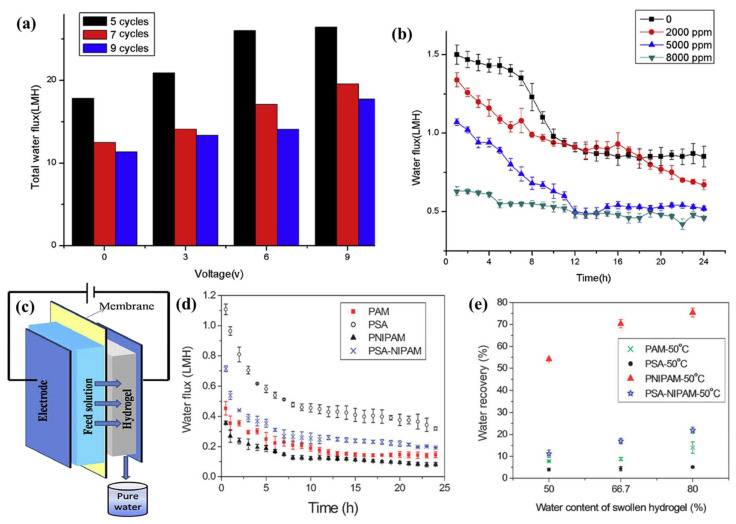
(**a**) Water flux of the as-prepared hydrogels over 24 h when different electric fields were applied; (**b**) water flux during 24 h FO using HA-PVA-5 hydrogel as traction agent. Different concentrations (2000, 5000, 8000 ppm) of deionized water and NaCl solutions were used as feed solutions; (**c**) schematic representation of an HA-PVA polymer hydrogel-FO desalination process [74]; (**d**) water flux during 24 h in FO process using polymer hydrogel and 2000 ppm NaCl solution as feed; (**e**) water recovery of swollen hydrogels (PAM, PSA, PNIPAM, and PSA-NIPAM) with different water loadings after dehydration at 50 °C for 2 min [75] (Reprinted with permission from Ref. [75] Li et al., 2011).

Polymer hydrogels are three-dimensional network structures cross-linked by polymer chains. A large number of hydrophilic groups make polymer hydrogels capable of absorbing a lot of water [83,84]. As a result, a hydrogel can play the role of a draw solution through direct contact with the membrane, without relying on water as a medium. An ideal hydrogel solute is characterized by a high swelling rate, low cost, and high mechanical strength [85]. Zhang et al. [74] were the first to evaluate the electro-responsive polymer hydrogels as draw agents in an FO process. A schematic representation of their FO desalination process employing HA-PVA polymer hydrogel as the draw agent is shown in Figure 12c. As shown in Figure 12a, the HA-PVA-5 polymer hydrogel (freeze–thaw cycles = five) produced the highest total water flux of 26.47 L/m^2^·h within 24 h when deionized water was used as the feed solution with a 9 V electric field. As shown in Figure 12b, the total water flux produced by the HA-PVA-5 polymer hydrogel becomes lower when NaCl is used as the feed solution, and reaches the highest total water flux of 22.66 L/m^2^·h in the 2000 ppm NaCl solution at 6 V. Their results indicated the high flux of the electro-responsive hydrogel. Moreover, the reverse salt flux of the draw solution was avoided and the process was greatly simplified by employing an electro-responsive hydrogel as the draw agent. Li et al. [75] used an ionic polymer hydrogel with thermally responsive units to obtain a high water permeation flux during an FO process, and more water was produced under the dual stimuli of pressure and heating. This process involved FO and dehydration. In the first step, the water permeated through the selective membrane, driven by the swelling pressure of the polymer hydrogel; the second step was dehydration of the swollen polymer hydrogel under various stimuli. Compared with the electro-responsive polymer hydrogels, the water flux of the thermally responsive polymer hydrogels performed worse. As shown in Figure 12d, the water fluxes of the four different types of polymer hydrogels were lower than 1 L/m^2^·h when 2000 ppm NaCl was used as the feed brine. Figure 12e shows that, when the swollen hydrogels were dehydrated under thermal stress conditions (50 °C), the water recovery was significantly improved. Overall, there are still many advantages and potentials for use for the hydrogel solutes as draw agents in the FO processes.

## 5. Advances in FO Membranes

**Figure 13:**
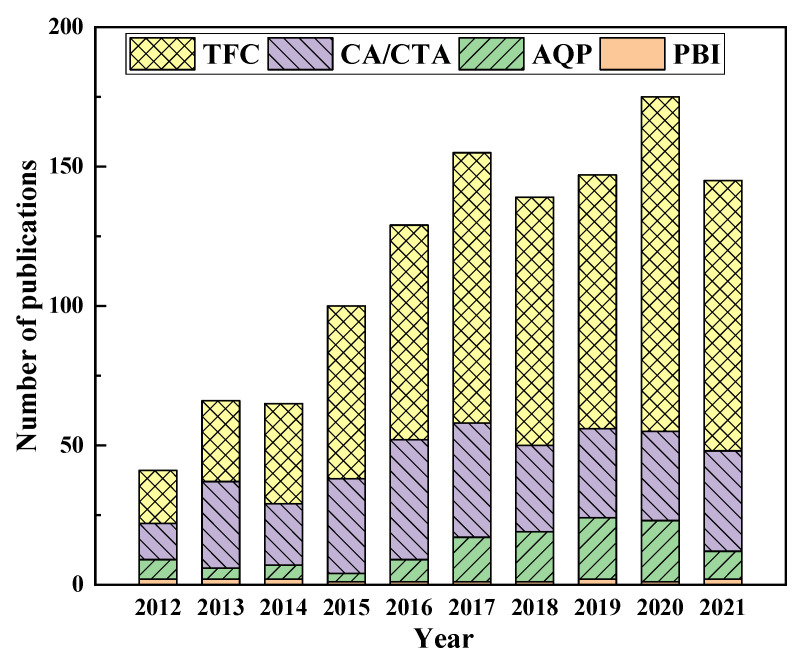
The numbers of publications on different FO membranes used in wastewater applications (data source: Scopus, 2012–2021).

The optimization of the membrane is a main focus in the FO separation processes, not least in improving the efficiency of the FO treatment of the highly saline wastewater so as to minimize membrane pollution. Currently, the study of the dense and homogeneous membrane materials remains practically very difficult, so most of the relevant research objects are still porous structures [86]. The research on FO membrane materials and structures is gradually gaining pace. This research on the high-performance FO membranes mainly concerns the development of new membrane materials, optimizing membrane active layers and/or support layers, and improving the water flux, solute retention capacities, anti-fouling abilities, and mechanical strengths of the membranes [87,88,89,90]. The emerging membranes can be classified into four categories, based on their fabrication methods, namely; those based on cellulose acetate (CA); thin-film composites (TFCs); polybenzimidazole (PBI); and aquaporin (AQP). The distribution of these membranes used in the FO wastewater treatment research is shown in Figure 13. It is clear that the TFCs have become the most competitive membranes.

### 5.1. CA Membranes

The CA membrane, a porous membrane material prepared by the acylation of cellulose with acetic anhydride, is widely used as an FO membrane, because of its favorable properties, such as good mechanical strength, wide availability, and relatively high hydrophilicity that endows it with a low fouling tendency and good water flux [91]. The cellulose triacetate (CTA) membrane has been the most commonly used CA membrane, and was the earliest commercial FO membrane used in the water treatment field [92]. For three decades from the 1990s, the CA membranes manufactured by HTI Co. were the only FO membranes produced on a full-scale production line. Only recently have other companies designed and produced new membranes on a large scale, but these membranes remain largely unapproachable for academic research [93]. Table 2 summarizes the fabrication of the CA FO membranes in the last ten years.

The preparation technologies for CA membranes continue to mature, and have been the subject of some research and analysis in recent years. Nguyen et al. [95] tested and compared the performances of their prepared CTA/CA membranes with those of the commercial membranes. A suitably composed FO membrane prepared under optimal preparation conditions showed a higher water flux and salt resistance than the commercial membranes. However, the performance of these membranes was not so high. In order to better explore the CA membranes, Zyaie et al. [97] fabricated a flat-sheet membrane with phase inversion by an immersion precipitation method and evaluated the effects of the CA concentration, bath temperature, and thermal annealing treatment on both the membrane structure and performance, using deionized water (working pressure 1 bar) passing through the feed channel and 1 M NaCl solution flowing through the draw channel. For an optimal membrane fabricated under the influence of annealing treatment with a CA concentration of 20 wt.% at a bath temperature of 23 °C, the water flux was 21.75 L/m^2^·h and the reverse solute flux was 5.88 g/m^2^·h. These authors observed the formation of finger-like macrovoids and a sponge-like porous structure at low CA concentration and high bath temperature, which promoted the enhancement of the water flux of the membrane.

**Figure 14:**
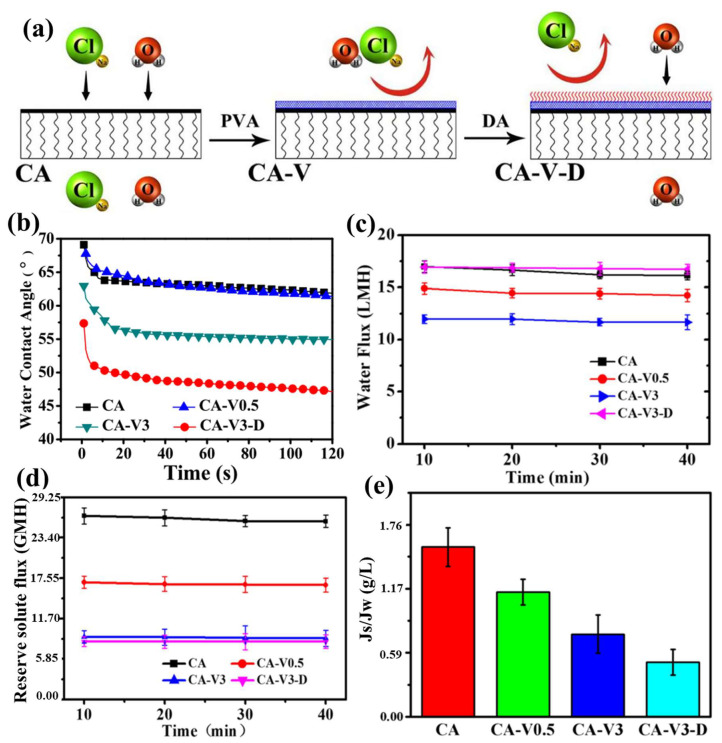
(**a**) Schematic diagram of CA FO membrane modification; (**b**) typical curves of water contact angle decaying with drop age; (**c**) water fluxes; (**d**) reverse salt fluxes; (**e**) J_s_/J_w_ (g/L) ratios [96] (Reprinted with permission from Ref. [96] Song et al., 2018).

The performance of a pristine CA membrane in solute separation is inadequate, and conventional modification methods usually involve multiple steps, which are not conducive to controlled preparation. Ahn et al. [94] fabricated polyvinyl alcohol (PVA)-coated CA-based flat-sheet membranes, using PVA as a surface modifying agent, and tested them for water flux and solute separation. Concentrated NaCl solution 1.5 M was used as draw solution and the feed solution used deionized water. The modified membrane exhibited a water flux of 8.8 L/m^2^·h, 20% higher than that of a pristine CA membrane (7.4 L/m^2^·h), without salt leakage, indicating that the PVA coating improved the water flux performance for the FO processes. Based on the above-mentioned improvement of a CA membrane by PVA, Song et al. [96] further modified a high-performance CA composite FO membrane with polydopamine (PDA). As shown in Figure 14a, PVA was first cross-linked on the surface of the CA membrane, and then the PDA was applied by a rapid deposition method. As shown in Figure 14b,e, the modified membrane coated with PDA and PVA, designated as CA-V3-D, exhibited the best hydrophilicity and showed a water flux of 16.72 L/m^2^·h and a reverse solute flux of 8.19 g/m^2^ h, with deionized water as the feed solution and 2.0 M NaCl as the draw solution. The obtained CA-V3-D performed better when its selective layer faced the feed side. The simple and efficient modification method greatly improved the performance of the CA membrane and made it an excellent candidate for the further study of the CA membranes.

**Figure 15:**
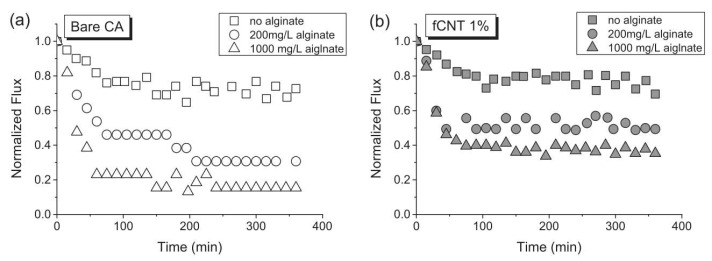
(**a**) Flux of bare CA membrane under different concentrations of alginates; (**b**) flux of 1% fCNT-CA membrane under different concentrations of alginates.

Compared with the PA-TFC RO membranes, the CA membranes are more hydrophilic and are more resistant to chlorine degradation, but their disadvantages, such as poor biological adhesion and hydrolysis inhibition, should be considered when developing FO membrane materials [85]. As mentioned above, Choi et al. [58] synthesized fCNT-CA membranes for FO processes through phase inversion. They found that the fCNTs possessed improved alginate fouling resistance (Figure 15a,b), which was attributed to the strong electrostatic repulsion between the membrane and the alginate fouling species. Furthermore, the fCNT-CA membranes proved to be more hydrophilic, due to the carboxylic acid groups on the surface of the fCNTs.

### 5.2. TFC Membranes

**Figure 16:**
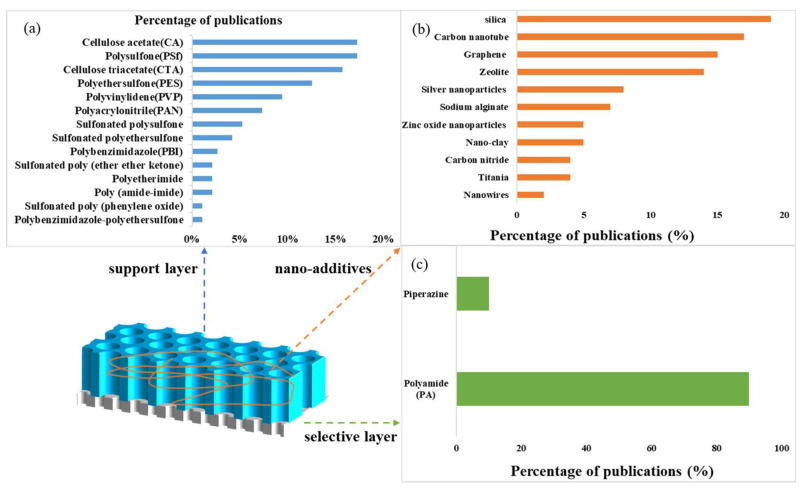
Percentages of publications on polymers and nano-additives used for flat-sheet FO membranes and hollow-fiber FO membranes: (**a**) support layer materials; (**b**) nano-additives; (**c**) selective layer materials (modified from [17,76]).

The preparation of the TFC membranes typically involves two steps: (i) a porous base film is prepared as a support layer by a phase inversion method; and (ii) an active layer is prepared on the surface of the support layer by an interfacial polymerization method [85]. The TFC membranes have become the most widely used membranes, due to their excellent characteristics, such as high water permeation flux, efficient solute rejection, and easy modification, whereby performance as an FO membrane can be optimized by adjusting the structure of either the active layer or the support layer. The percentages of publications on polymers and nano-additives used for flat-sheet FO membranes and hollow-fiber FO membranes are shown in Figure 16. The TFC FO membrane fabrication in the last ten years is shown in Table 3, from which it is evident that the polyamide (PA)-type TFC membranes have been most widely studied.

The efforts to improve the TFC membranes often concern two aspects, namely, the substrate structure and the hydrophilicity [112,113]. Wei et al. [90] prepared PA-TFC FO membranes with a tailored substrate structure by first producing PSf substrates with a finger-like pore structure by phase inversion, and then synthesizing polyamide active layers by interfacial polymerization. Comparing the performance of the synthesized TFC membranes with those of the commercial membranes, the critical importance of the support structure emerged, that is, the straight finger-like pore structure is superior to a sponge-like structure, since it can reduce the concentration polarization. When the active layer faces the draw solution, water fluxes as high as 54 L/m^2^·h can be achieved, using the 2 M NaCl as a draw solution, while maintaining relatively low reverse solute fluxes. By varying the conditions used during the production of the PSf layer, Tiraferri et al. [98] produced a range of substrates with differing structures, and proceeded to systematically study the effect of the substrate structure on FO performance. By using a 1 M NaCl draw solution and a deionized water feed, high water fluxes (up to 25 L/m^2^·h) and high solute rejections (>95.5%) consistently, confirmed the hypothesis that a mixed-structure substrate, with a thin sponge-like layer on top of a highly porous layer with macrovoids, constitutes an excellent TFC FO membrane. Furthermore, the membranes with a high water permeability flux and excellent selectivity are preferred to achieve both a high FO water flux and low solute flux.

Hydrophilicity is the key to achieving a high water flux within the substrate of an asymmetric membrane. Arena et al. [99] modified two commercially available TFC RO membranes (BW30 and SW30-XLE membranes) to improve their hydrophilicity. The modification method involved the use of PDA to coat the support layer, which not only resulted in increased hydrophilicity and a corresponding increase in “wetted porosity”, but also suppressed the internal concentration polarization. Their results showed that the water flux performances of the modified membranes exhibited 8–15-fold increases compared to the control data using a NaCl draw solution at 0.05, 0.1, 0.5, 1.0, and 1.5 M concentration, which indicated that this modification method could potentially render existing TFC membranes suitable for engineered permeation applications. Meanwhile, the water flux performances showed a two-fold increase for the SW30-XLE, but a reduction for the BW30 under the tested conditions. As mentioned in Section 2.2, Fang et al. [36] fabricated novel ultrafiltration–adsorption membranes for the adsorption of heavy metal ions, by decorating the inner pore walls of a PES membrane with PDA nanoparticles. The obtained PES/PDA membranes showed significantly enhanced antifouling performance, superior ultrafiltration efficiency, and a high adsorption capacity for heavy metal ions. This novel approach paves the way for the design and fabrication of other highly hydrophilic TFC membranes by incorporating PDA, which might be used to meet specific water treatment requirements.

The incorporation of TiO_2_ nanoparticles can also effectively enhance the hydrophilicity and porosity of FO membranes. Emadzadeh et al. [102] fabricated a range of PSf-TiO_2_ nanocomposite support layers by adding different amounts of TiO_2_ nanoparticles to a PSf substrate. Table 3 shows that the addition of the TiO_2_ nanoparticles increased the hydrophilicity and porosity of the substrate, and, by increasing the TiO_2_ loading, generated long finger-like structures to enhance the water permeability. In order to prepare a TFN membrane, a thin PA layer was formed on the upper surface of a PSf-TiO_2_ nanocomposite substrate by interfacial polymerization, which could potentially enhance water flux by 86–93% and reduce the reverse solute flux (10 mM NaCl concentration in the feed solution; 0.5 and 2.0 M NaCl concentration in the draw solution), showing great potential. It can thus be concluded that incorporating an appropriate amount of TiO_2_ nanoparticles into a PSf matrix may greatly enhance the performance of the TFN membranes in FO applications.

Investigations have shown that the hydrophobic support layers of the TFC membranes can result in severe internal concentration polarization, due to incomplete wetting, which decreases the effective porosity of the substrate, resisting material transport, and thus, water flux. Huang et al. [101] prepared a novel TFC membrane by depositing a PA selective layer on top of a nylon 6,6 microfiltration membrane substrate by interfacial polymerization. Compared with a commercial CTA FO membrane from HTI Co., the newly developed PA-TFC membrane showed comparable water flux, but its solute flux was only one-tenth of that of the commercial product, and its specific salt flux was only one-twenty-eighth of that of the HTI product (2000 ppm NaCl and 2000 ppm MgSO_4_ as the feed; cross-flow velocity of 0.26 m/s; 25 °C). The excellent performance of the TFC membranes in the permeation flux testing is entirely attributable to the high permselectivity of the active layers, and the hydrophilicity of the nylon 6,6 substrate. This suggests that these TFC membranes with a nylon 6,6 support layer could further enable applications, such as FO or PRO. In addition, the studies have shown the great potential of TFC hollow-fiber membranes for permeation processes. Ren et al. [103] prepared a TFC membrane with an inherently hydrophilic polyacrylonitrile (PAN) hollow-fiber support. A selective PA active layer was synthesized on the membrane shell side by interfacial polymerization. When 1.0 M NaCl was used as the draw solution in PRO mode, a water flux as high as 36.6 L/m^2^·h demonstrated the potential of utilizing inherently hydrophilic polymeric hollow fibers with fine-tuned pore structures as the TFC membrane supports.

The incorporation of NaY zeolite nanoparticles into the PA selective layer can also markedly improve the membrane separation performance. Ma et al. [100] were the first to propose the fabrication of zeolite-PA-based TFN membranes for FO applications, which were fabricated on PSf porous support layers tailored for the FO membrane separation process. The porous nature of the zeolite endowed the membranes with greatly enhanced water permeability. In all of the cases evaluated in their study (0.5–2.0 NaCl draw solution, deionized water and 10 mM NaCl feed solution, and both membrane orientations), the TFN membranes with a zeolite loading of 0.1 wt./v% were the most permeable; about 80% higher than the water permeability of the baseline TFC membrane. The successful fabrication and efficient utilization of the zeolite-PA-based TFN membranes provide an additional dimension and new opportunities for optimizing and improving the FO membrane performance, which deserves further attention from the FO research community.

**Figure 17:**
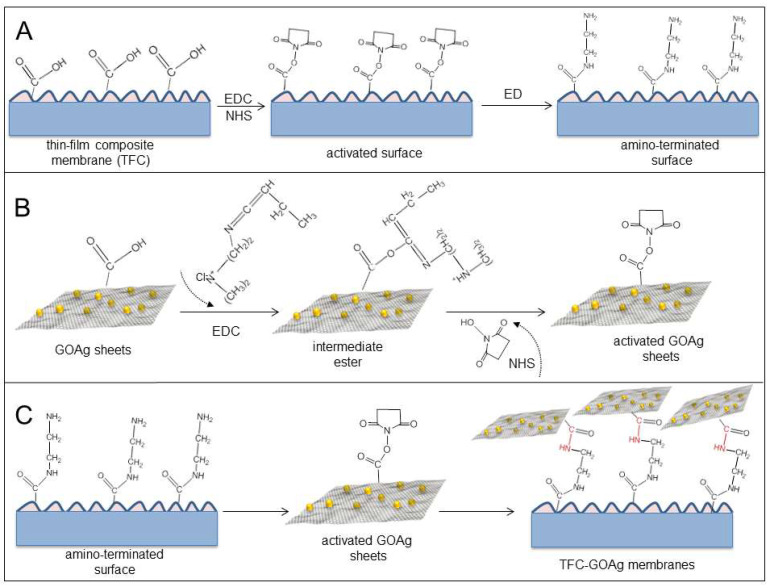
Scheme illustrating the three sequential steps for binding GO/Ag sheets to the surface of TFC membranes: (**A**) formation of an amine-terminated surface; (**B**) activation of carboxylic functional groups on GO/Ag sheets; (**C**) binding of GO sheets through the formation of amide bonds [104] (Reprinted with permission from Ref. [104] Faria et al., 2017).

The addition of GO can improve the competitiveness of the prepared PA-TFC membranes, by changing the structure and hydrophilicity of the active layer. Eslah et al. [88] explored the synthesis of the GO-incorporated PA-TFN membranes on a PSf substrate for FO applications, and found that the GO nanosheets altered the PA surface; increasing the loading of GO enhanced the surface hydrophilicity. The water flux of a TFN membrane with 0.1 wt.% GO embedded was improved from 7.9 to 14.5 L/m^2^·h, using deionized water as the feed solution and 1.0 M NaCl as the draw solution in FO mode, an improvement of 83.5% compared to the TFC, while the reverse solute flux was reduced by up to 39%. Li et al. [108] prepared GO-incorporated PA-TFN membranes in a similar way, and proceeded to investigate their resistance to chlorine. The water flux of the GO-3 PA-TFN membrane increased 56.97% using the 2.0 M NaCl draw solution against deionized water, at the same time, these membranes showed up to 75.0-fold higher chlorine resistance than the control group, offering a good desalination performance. Zhao et al. [106] explored a method of inhibiting concentration polarization and improving separation performance, by applying an intermediate layer of GO and multi-walled carbon nanotubes (MWCNTs). A novel GO/MWCNT TFN membrane was prepared by synthesizing a GO/MWCNT intermediate layer on a hydrophilic nylon 6,6 microfiltration substrate by interfacial polymerization. The synthesized GO/MWCNT intermediate layer not only provided nanochannels for faster water transport, but also reduced the PA layer thickness by 60%. Through this treatment, the water flux of 30.0 L/m^2^·h was effectively increased by 60% and the reverse solute flux of only 5.02 g/m^2^·h was decreased by 50%, compared to a membrane without a GO/MWCNT composite layer (with 1.5 M NaCl as the draw solution). In general, GO-modified TFN has good water permeability, salt interception, and chlorine resistance, and has been developing well. To the explore bacterial adhesion prevention properties, Faria et al. [104] fabricated TFN membranes, functionalized with a GO/Ag nanocomposite. Figure 17 illustrates the three sequential steps whereby the GO/Ag sheets were bound to the surface of a TFC membrane, in which the carboxyl groups on the GO/Ag nanosheets were covalently bonded to the carboxyl groups on the surface of the TFC membranes through a cross-linking reaction. The immobilization of the GO/Ag nanocomposite on the membrane surface was confirmed by further characterization. When the deionized water and NaCl solutions were used as the feed and the draw solutions, respectively, the results presented showed that the GO/Ag modified membranes exhibited an 80% inactivation rate against the attached pseudomonas aeruginosa cells, but the water flux presented a small decrease from 5.12 to 4.67 L/m^2^·h·bar and the salt permeability of 1.59 L/m^2^·h still remained low. A series of experiments showed that functionalization with the GO/Ag nanocomposite exhibited excellent biofouling resistance, without sacrificing the inherent transport properties of the membranes.

**Figure 18:**
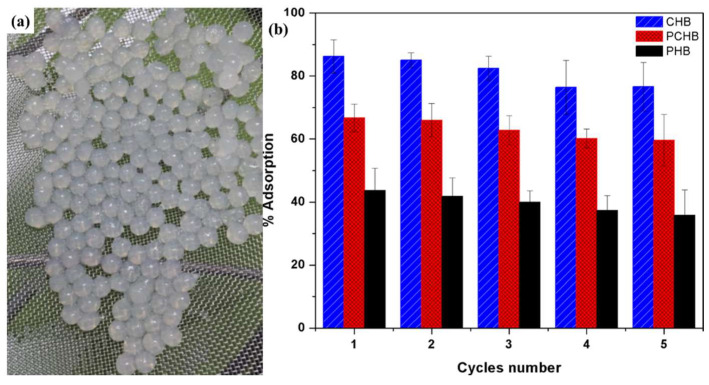
(**a**) Appearance of hydrogel beads; (**b**) reusability of the adsorption capacity of hydrogel beads [114] (Reprinted with permission from Ref. [114] Jamnongkan et al., 2021).

Hydrogel beads are an efficient and reusable adsorbent for the removal of heavy metal ions from aqueous solutions. Based on the biomaterials shown in Figure 18, Jamnongkan et al. [114] fabricated novel hydrogel beads using chitosan, PVA, and their blend for copper (II) ion removal from aqueous solution (named CHB, PCHB, PHB, respectively). The results showed that the CHB has the highest copper ion adsorption rate of more than 80%. Their reusability study, as shown in Figure 18b, showed that the hydrogel beads have stable reusability, among which CHB has the best performance. Owing to the desirable properties of both the PVA and GO flakes as a membrane coating, Akther et al. [110] used GO-doped PVA hydrogel as the material with which to coat the PA layer of the commercial TFC membrane, to enhance the FO performance. The PVA hydrogel coating-modified the TFC membranes with 0.02 wt.% GO, that showed a 55% reduction in the specific reverse solute flux and only a slight decrease in the water flux, while the best antifouling performance was improved 58%. The bactericidal GO flakes in the PVA hydrogel coating also enhanced the biofouling resistance of the modified membrane, which can be attributed to its morphology and superior surface properties.

The TFC membranes have also contributed significantly to the treatment of many types of polluted wastewater generated by industry. To explore the fouling resistance performance of the TFC FO membranes, Alireza et al. [105] synthesized the TFC membranes with different concentrations of PSf and applied them for the removal of two TrOCs (benzene and phenol) from aqueous solutions. Their results showed that the TFC membranes incorporating 16% and 17% PSf exhibited the highest efficiencies, and that increasing the concentration of the draw solution could remove more of the phenol and benzene. The highest removal efficiencies for the phenol (polar) and benzene (nonpolar) were 79% and 90%, respectively, showing that the TFC membranes had an excellent ability to remove the TrOCs from aqueous solutions under various process conditions, with the removal of the nonpolar compounds being most favored. Alireza et al. [109] also synthesized TFN membranes and evaluated their efficacy for the removal of three TrOCs from synthetic and real industrial wastewater samples. The substrate of the TFN membrane was composed of polyethylene glycol 400 (PEG-400), PSf, and 1-methyl-2-pyrrolidone, mixed in various ratios by phase inversion, while the selective layer of the TFN membrane was composed of GO, *m*-phenylenediamine, and 1,3,5-trichlorobenzene, mixed in various ratios by interfacial polymerization. A TFN membrane with 0.008% GO in the selective layer and 4% PEG-400 in the substrate exhibited the highest water flux (34.3 L/m^2^·h) and the highest removal efficiencies for benzene, phenol, and toluene (97, 84, and 91%, respectively), revealing the promising potential of such TFN membranes in improving the membrane separation performance and removing TrOCs from wastewater.

### 5.3. PBI Membranes

Polybenzimidazoles (PBIs), a class of heterocyclic polymers, were originally developed by Celanese in 1983, of which poly-2,2′-(*m*-phenylene)-5,5′-bibenzimidazole (PBI) is the most widely studied. The PBI membranes, due to their excellent chemical, thermal, and mechanical stabilities, have been widely used in the RO and NF processes and as ion-exchange membranes for fuel cells [115]. Table 4 shows the development status of PBIs in FO in recent years.

**Figure 19:**
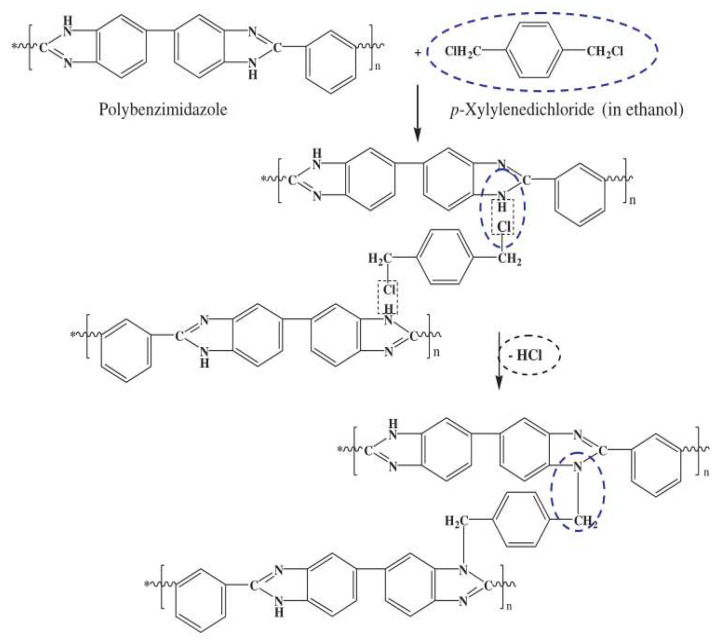
Possible mechanism of *p*-xylene dichloride-modification of PBI [117] (Reprinted with permission from Ref. [117] Wang et al., 2009).

Wang et al. [116] first investigated the potential of the PBI NF membranes for FO. The main reasons for choosing PBI for FO were its excellent NF properties, high mechanical strength, and unique chemical stability. A series of water flux tests employing different concentrations of MgCl_2_, MgSO_4_, Na_2_SO_4_, and NaCl solutions as draw solutions and deionized water as the feed solution showed that the membrane exhibited a high water permeation flux (11.2 L/m^2^·h) and an excellent salt selectivity (99.79%) when 5.0 M MgCl_2_ solution was used as the draw in PRO mode. The PBI NF membranes with narrow pore size distributions were particularly promising candidates for FO membranes. In subsequent research, Wang et al. [117] further fabricated a series of hollow-fiber PBI NF membranes with thin walls and desired pore sizes through non-solvent-induced phase inversion and chemical cross-linking modification. Figure 19 shows the possible mechanism of the *p*-xylene dichloride modification of PBI. Compared with the previous research data on PBI membranes [116], the water flux of the newly developed PBI NF membrane was improved from 11.2 to 36.5 L/m^2^·h, using 5.0 M MgCl_2_ solution as the draw solution and deionized water as the feed solution. Moreover, a PBI NF membrane modified for 2 h, with high permeation flux (36.5 L/m^2^·h) and improved salt selectivity (99.5%) in PRO mode, could be used for water recovery from wastewater, whereas a longer cross-linking time rendered the PBI membrane more suitable for seawater desalination, further demonstrating that the hollow-fiber PBI NF membranes had good permeability. The continuous researches by Wang et al. have shown the great potential of PBI in water treatment performance. Future research on PBI will further optimize the NF hollow fiber structure to reduce the internal concentration polarization and study its performance in more practical applications.

The significant drawbacks of PBIs, such as hydrophobicity and net zero surface charge at neutral pH, have prevented their use as membranes for osmosis applications. The surface functionalization has been extensively studied as a means of overcoming the above drawbacks by modifying the PBI membranes to increase their surface charge and enhance their hydrophilicity. Flanagan [118] fabricated and investigated functionalized asymmetric flat-sheet PBI membranes, which exhibited enhanced hydrophilicity, increased surface charge, and reduced pore size when testing a 2 M ammonium bicarbonate solution against the feed solutions of 0.1 M sodium chloride solutions in FO mode. All of the modified membranes showed an increased water flux from 4.1 to 5.7 L/m^2^·h and decreased NaCl transport from 4.5% to 1.5%. However, the thermal stability of the above-mentioned membranes was less than satisfactory. Daer et al. [123] prepared flat-sheet FO membranes by phase separation from PBI-doped solutions with different loadings (0, 0.5, 1, and 2 wt.%) of silica nanoparticles. The addition of the silica nanoparticles to the PBI membranes reduced their structural parameters, augmented their tensile strength, and doubled their water flux (16.9 L/m^2^·h) compared to a PBI membrane control group (7.4 L/m^2^·h) (testing conditions: 2 M NaCl draw solution; deionized water feed solution; cross-flow velocity, 2 cm/s; the active layer facing the draw solution). Considering the thermal stability of the PBI/silica membrane, coupled with its improved water permeation performance, the modified membrane offers promise for FO processes in hot and arid zones. To further optimize the membrane performance, Fu et al. [120] developed a novel mixed-matrix hollow-fiber membrane, with an outer layer composed of PBI and polyhedral oligomeric silsesquioxane (POSS) and an inner layer composed of polyacrylonitrile (PAN) and polyvinylpyrrolidone (PVP). The incorporation of POSS and PVP into the outer PBI and inner PAN, respectively, resulted in the formation of an integrally macropore-free and delamination-resistant dual-layer membrane. Moreover, the increase in the POSS concentration in the PBI simultaneously enhanced the water flux and salt permeability across the membrane. The results showed that the membrane with this optimized concentration showed a maximum water flux 31.37 L/m^2^·h at room temperature, using 2.0 M MgCl_2_ as the draw solution in the FO process, and the membrane performance achieved a great improvement. Its excellent fully hydrophilic structure, easy processability, and cost-effective ultra-thin PBI outer layer may enable the novel membrane to be widely applied in the future.

To develop the next-generation high-performance semi-permeable membranes, Aiba et al. [122] synthesized and characterized novel semi-permeable membranes, based on cross-linked PBI. The cross-linking reaction of PBI with *n*-butyl sulfonate and divinyl sulfone modified the pore size distribution, such that the selective permeation of water molecules was successfully achieved. When a 500 mg/L aqueous solution of NaCl was supplied to the membranes, the flux of the newly developed PBI NF membranes was increased from 1.88 to 22.1 L/m^2^·h, and the NaCl rejection was improved from 11% to 46% by the facile cross-linking reaction, expediting the development of high-performance semi-permeable membranes for water treatment, including by the RO, FO, and PRO.

**Figure 20:**
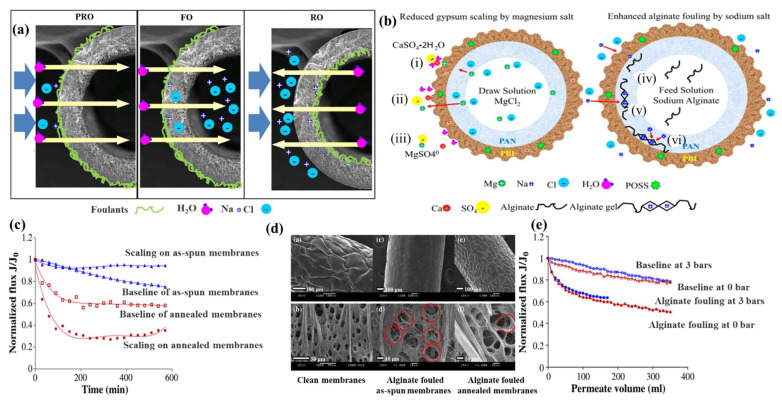
(**a**) Fouling mechanisms in PRO, FO, and RO processes; (**b**) Schematic illustration of MgCl_2_ influence on gypsum fouling and NaCl effects on alginate scaling in FO mode: (i) diffusion of Mg^2+^; (ii) competition between Ca^2+^ and Mg^2+^ for SO_4_^2−^; (iii) formation of the MgSO_4_ complex; (iv) diffusion of Na^+^, (v) formation of alginate gel; and (vi) competition of Na^+^ for an adsorption site of alginate; (**c**) Normalized flux J/J_0_ for baseline experiments and gypsum fouling of as-spun and annealed PBI-POSS/PAN membranes (testing conditions: 70 mM CaCl_2_, 38 mM Na_2_SO_4_, 40 mM NaCl scaling solution (shell side, PBI layer), MgCl_2_ draw solution (lumen side, PAN layer)); (**d**) SEM images of PBI-POSS/PAN membranes with different treatments; (**e**) Normalized flux J/J_0_ for baseline experiments and alginate scaling on annealed PBI-POSS/PAN membranes (testing conditions: 1.60 M NaCl draw solution) [121] (Reprinted with permission from Ref. [121] Chen et al., 2014).

PBI is very prominent in the treatment of fouling. By investigating the FO processes of CA, PBI/PES, and a newly developed PBI-POSS/PAN membrane, Chen et al. [119] were the first to find that surface ionic interactions play the dominant role in gypsum fouling on a membrane surface. Strong attractive forces led to a 70% reduction in the flux on negatively charged CA and PBI membrane surfaces. The PBI-POSS/PAN membrane showed a ridged morphology and a contact angle of 51.42° ± 14.85° after the addition of hydrophilic POSS nanoparticles and thermal treatment at 95 °C for 3 min. Minimal fouling and a flux reduction of only 1.3% were achieved at pH 3, at which such a ridged morphology could reduce fouling by not providing a locally flat surface for crystallite deposition; thus, any gypsum on the surface would be easily washed away. In further research on as-spun and annealed PBI-POSS/PAN hollow-fiber membranes, Chen et al. [121] explored the individual influences of the water permeation flux and reverse solute flux on the scaling behavior of these membranes in the PRO, FO, and RO processes (Figure 20a). The gypsum fouling (inorganic scaling) in the FO process and sodium alginate fouling (organic scaling) in the PRO process were investigated. It is shown in Figure 20b that the competitive formation of the MgSO_4_ and gypsum increased the reverse MgCl_2_ flux, which could inhibit and even eliminate the gypsum fouling on the membrane surface. As shown in Figure 20c, a large flux reduction of about 70% was observed on the annealed membranes, but a slight flux reduction of 6% was observed on the as-spun membranes. In the SEM image in Figure 20d, significant alginate contamination can be seen on the outermost surface of the as-spun membrane. Figure 20e illustrates that the fouling in PRO mode was slightly lower than in FO mode (36% vs. 40% flux reduction). A comparison between Figure 20c,e indicates that increasing the reverse NaCl flux slightly enhanced the alginate fouling. The water permeation flux, rather than the reverse solute flux, always plays the dominant role in scaling. Thus, reducing the initial flux in the FO process may alleviate the contamination phenomenon more significantly than increasing the reverse NaCl flux.

### 5.4. AQP Membranes

Aquaporin (AQP) membrane, a transmembrane protein with extremely high selectivity and permeability to water molecules, is fabricated by directly or indirectly embedding AQP in the organic matrix membranes (such as those for NF, RO, and FO). The selective permeability of the AQP can achieve a higher water flux and solute interception [124,125].

In recent years, FO, as a promising alternative to the traditional pressure-driven filtration, has attracted widespread attention in removing trace organic matter from water. Li et al. [126] incorporated AQP into a PA selective layer, which remarkably enhanced its water permeability flux from 25.4 to 38.5 L/m^2^·h. The FO membrane incorporating AQP outperformed most of the other reported FO membranes.

**Figure 21:**
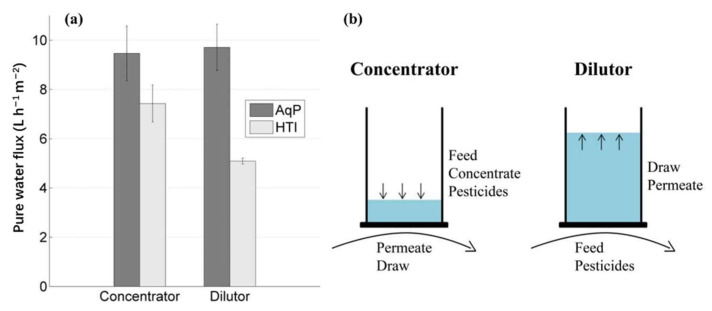
(**a**) Comparison of fluxes and (**b**) illustrations of set-ups for the concentrator and dilutor modes [128] (Reprinted with permission from Ref. [128] Madsen et al., 2015).

The FO membranes in current use perform poorly in retaining the small molecules of neutral organic pollutants, which limits their applicability in FO processes. However, AQP can help FO solve this problem. As described in Section 3.2, Luo et al. [59] investigated the removal of 30 TrOCs by a novel biomimetic AQP FO membrane in an osmotic membrane bioreactor. Their results showed that all of the 30 selected TrOCs were more than 85% removed, regardless of their diverse properties, indicating the stability and compatibility of the AQP membrane in combination with the activated sludge treatment. However, the thermal stability of the film was not mentioned in their researches. In the study of Xie et al. [127], the transport mechanisms of TrOCs through an AQP-TFC membrane were explored again, and the membrane stability with respect to TrOC rejection under extreme conditions was further verified. The newly fabricated AQP FO membrane displayed superior contaminant rejection, achieving more than 90% inhibition of all of the studied TrOCs, using 2 M NaCl as the draw solution. The TrOC transport was found to be dominated by the solution-diffusion mechanism, and the dominant transport resistance could be attributed to the PA matrix rather than to the AQP vesicles. In addition, the AQP membranes showed a good thermal stability, such that the organic contaminant rejections before and after heat treatment were essentially the same, whereas contact with the ethanol compromised membrane performance. However, the removal effect of the TrOCs was still not high. In order to further improve the removal efficiency of the TrOCs, Madsen et al. [128] developed a novel AQP membrane for the removal of three selected TrOCs that could be considered as benchmarks for the ability of a membrane to retain small neutral organic contaminants. It can be seen in Figure 21a that the AQP membrane facilitated a statistically significant higher pure water flux than the HTI membrane. Figure 21b illustrates how the system may be operated in two modes: concentrator or dilutor mode. The AQP membrane showed rejection values above 97% for all three of the TrOCs, higher than those in all of the previous studies.

Iodide is a precious substance, whereas boron is a toxic material with an emission limit of 1 mg/L in Taiwan. For the first time, Chang et al. [107] utilized FO technology to simultaneously recover iodide and remove boron from thin-film transistor liquid-crystal-display wastewater. The CTA and AQP-TFC were tested comparatively under different conditions, and the latter exhibited a high boron recovery of 98.4% and an iodide rejection of 98.3% at pH 11. In addition, the iodide recovery and boron rejection efficiency were both enhanced to 99.9% by using 0.5 mM cetyltrimethylammonium bromide as the draw solution. The boron that remained in the draw solution was as low as 0.64 mg boron/L. Thus, this FO system, coupled with the membrane distillation process used to concentrate and purify the MgCl_2_ draw solution, may be applied for iodide recovery and boron removal in the thin-film transistor liquid-crystal-display industry.

## 6. Sustainability of FO Water Treatment

As reviewed above, the research activities on FO water treatment are focused on the draw solution selection, membrane fabrication and modification, and membrane fouling. The FO processes have demonstrated their advantages and potential in treating wastewater and salty water, including low energy consumption through membranes, reversible membrane fouling, and low operating costs. Nevertheless, FO is not yet a mature technology, and remains far from reaching full expectations. It is recognized that FO water treatment still faces some sustainability challenges related to low-cost and energy-efficient regeneration processes [4].

### 6.1. Continuous Development of High-Tech FO Membranes

The development of high-flux FO membranes continues apace for FO water treatment. An ideal high-flux FO membrane should have the following characteristics: (i) a material showing excellent salt rejection and water flux; (ii) a large area for improved FO process efficiency; and (iii) a mechanically strong supporting membrane to withstand hydraulic pressure to extend working life. In the 1990s, the first commercial FO membrane, developed and produced by HTI Co., was made of CTA and consisted only of an active layer and a porous support layer; it was 93 μm thick and was made by the phase inversion method. Since then, increasingly diverse FO membranes have been developed, among which hollow-fiber membranes and bionic fiber membranes may have great potential in the coming years. Hollow-fiber membranes are widely used in the osmosis processes, due to their low production costs and self-supporting capabilities. Compared with flat-sheet membranes, the hollow-fiber membranes can provide a high osmosis area-to-volume ratio, thereby obtaining high permeation fluxes from a smaller membrane area, which raises their profile for industrial applications [4,103,129].

Based on biomimetic membrane technology, Madsen et al. [128] tested a newly developed AQP membrane for the removal of three selected TrOCs. AQP is a pore-forming protein that, when combined with a membrane structure, can promote water diffusion under the action of an osmotic pressure gradient. Compared with traditional membranes, AQP has higher permeability. Some AQP membranes, such as GlpF, are permeable to molecules other than water, and can transport glycerol, arsenite, urea, and glycine, while other AQPs, such as AqpZ, can only transport water [53]. In the context of the removal of TrOCs, it is meaningful to combine high permeability with selective transport, because it is possible to obtain a dense active layer with high permeability without affecting the flux, even for small molecules of neutral trace organic matter [130,131]. However, for the sustainable development of biomimetic membranes, the issues of AQP biodegradation, the effect of chemical cleaning, and applicability at different pH and temperatures, need to be addressed.

### 6.2. Long-Term Exploration of Draw Solutions

For the FO water treatment, further novel and durable high-performing draw solutions are needed to ensure the continuous operation of the FO system and to reduce energy consumption. Gaseous, inorganic-based, and bionic draw solutes have great future prospects. Some volatile solutes, such as ammonium hydrogencarbonate and ethanol, have the potential to be used as the draw solutes to extract water from a saline feed solution across a semi-permeable polymeric membrane. After moderate heating, the gaseous substances (such as ammonia, carbon dioxide, and ethanol vapor) are separated and recycled, leaving the fresh product, water. The gaseous solutes are more suitable for the treatment of low concentration salt wastewater due to their lower osmotic pressure and lower energy requirements for separation and recovery. Fertilizers are also a good choice as the draw agents. The diluted fertilizer draw solution may be directly used for fertilization, rather than being separated, and the energy cost of this process merely includes the power input of pumping, storage, and so on; it may be widely used in agriculture. Inorganic salts have high water flux, but they cannot avoid the shortcomings of the reverse solute flux with small molecules. Finding ways to reduce the reverse solute flux in the FO process is a core issue when screening and optimizing the draw solutes [17]. Besides, the bionic draw solutes show great potential. Many of the proteins present in animals, such as BSA [132], have good selective permeability and represent a potential new class of FO draw solutes. By combining them with MNPs, novel and smart FO draw solutes may be developed. The continued development of draw solutions that can provide high osmolarity is another critical step towards further progress in the FO processes.

### 6.3. Ongoing Research on Fouling

Fouling is an enormous challenge to many membrane separation technologies and applications. The FO water treatment technology has been extensively studied due to its advantage of a low membrane fouling tendency. Nevertheless, sustainable water treatment still remains a daunting task, because of the complex pollutants in the highly saline wastewater and seawater. Thus, there is an urgent need to explore commonly applicable antifouling FO membranes. The membranes prepared from nanomaterials may gradually become some of the most widely used antifouling membranes for sustainable FO water treatment. With the steady development of nanotechnology, the nanomaterials, such as nanoparticles [102], carbon nanotubes [106] and GO [104], have been widely used in the preparation of FO membranes. They may be integrated into membranes or deposited on membrane surfaces, so as to achieve antifouling performance. Further attention should be directed towards developing novel nanomaterials, probing their nanostructures, and analyzing how well they can be integrated into FO membranes. In addition, some multi-dimensional nanomaterials, such as metal–organic frameworks, should be evaluated as building blocks for permeable membranes, due to their excellent antifouling properties. Last but not least, biomimetic technology remains a focal point in our antifouling research. We may derive new antifouling ideas from the structures and functionalities of the naturally strong antifouling surfaces of plants (e.g., lotus and rice leaves) and animals (e.g., fish scales, shark skins, cicada wings, and gecko feet) for the sustainable water treatment technology for the defense of antifouling films [133].

### 6.4. Reasonable Utilization of Hybrid Systems

**Figure 22:**
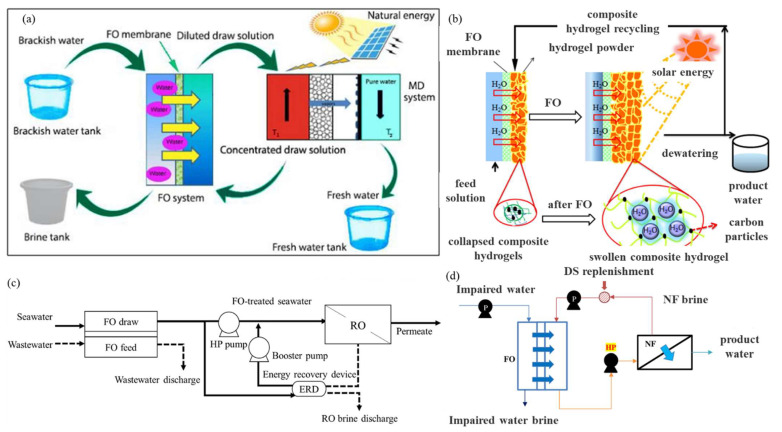
Schematic illustration of hybrid FO systems: (**a**) a solar energy-driven system for brackish water desalination and fertilizer [137] (Reprinted with permission from Ref. [137] Suwaileh et al., 2019); (**b**) a solar energy-driven hydrogel recycling process for desalination [136]; (**c**) a hybrid desalination system of FO-RO [138]; and (**d**) a hybrid desalination system of FO-NF [139].

It is also necessary to apply a second separation step to obtain high-quality permeated water, which may be suitably accomplished by relying on green energy sources (for instance, natural energy, industrial waste heat, geothermal energy, or biomass energy). The FO process offers a very promising solution for the treatment of different effluents (e.g., wastewater and brackish water), and generates product water with different qualities (e.g., reduced brine and clean water) for non-potable water reuse purposes. There is great potential for the gaseous draw solutes, inorganic-based draw solutes, and MNP draw solutes to produce high-quality permeate water. In the second separation step, the removal of the gaseous draw solutes requires heating, the removal of the inorganic-based draw solutes requires a complicated crystallization process, and the removal of the MNP draw solutes needs a strong magnetic field [134,135]. A hybrid system combined with other energy sources, for instance, renewable energy (wind energy or solar power), industrial waste heat, geothermal energy, or biomass energy (Figure 22a,b) [136,137], may be used to minimize the energy consumption and operating costs arising from the second recovery system, thereby promoting the sustainable application of FO.

Several water treatment processes [140]: adsorption and advanced oxidation processes (photodegradation, photocatalysis, ozonation, Fenton reaction, Wet Air Oxidation, ultraviolet radiation, hydrogen peroxide oxidation), are combined with FO; their performance in the water treatment process will be of great importance to the future development of FO. FO-adsorption could be a hybrid system [141] of an FO process and a physical adsorption process, using industrial waste or activated carbon as an adsorbent, could also be an FO process [142,143], in which the FO membrane is prepared using an adsorbent material for better permeation. A novel membrane photocatalyst [144] will perform well in water treatment by integrating photocatalysis and FO process. The FO-MF [145], FO-NF [139], and FO-RO [138] hybrid systems, as shown in Figure 22c,d, due to this relatively low feed water concentration, can also be expected to greatly reduce the energy consumption and obtain high-quality water. Other hybrid systems, such as FO-photodegradation, FO-ozonation, FO-hydrogen peroxide oxidation, and FO-membrane bioreactor, hold great promise for the removal of salinity and organics from water through FO.

## 7. Conclusions

In recent years, FO, an evolving and cost-effective membrane separation technology, has emerged as a promising alternative to the traditional water treatment approaches (such as NF and RO). The aim of this review has been to provide an objective and comprehensive evaluation of the current trends, highlighting advances in the draw solutes, membranes, and sustainability, whilst also highlighting the issues that have hampered the large-scale deployment of FO technology in water treatment.

The membrane material and the draw solute are the two key factors that need to be optimized to maximize the process efficiency for the FO water treatment. The most desirable FO membranes would be chemically and mechanically stable with features ensuring maximal water flux, minimal internal concentration polarization, and negligible reverse solute flux. It is preferable to fabricate a high-density porous support layer to reduce the internal concentration polarization and a highly selective active layer to inhibit the reverse solute diffusion. The draw solutes for the future FO treatment wastewater applications will be those that can provide the maximum osmotic pressure, while requiring minimal energy for their regeneration. FO membrane fouling would be reduced if the support layer could restrict the reverse flux of the draw solutes. Using small molecules/ions as the draw solutes will minimize the internal concentration polarization, but will also increase the reverse solute flux. Therefore, it is very important to minimize the reverse migration of the draw solute, while maintaining the high water flux in the forward direction. In summary, it can be concluded that the characteristics of the solute and the membrane essentially determine the internal concentration polarization, the membrane fouling, and the reverse solute flux. A hybrid system, combined with other energy sources or other membrane separation technologies, could be used to minimize the energy consumption and operating costs associated with the second recovery system, which will promote the sustainable application of FO.

## Figures and Tables

**Table 1:** Several common extracted solutes in recent years.

Year	Draw Solutes/Solutions	Type	FO Performance	References
2015	Ammonium hydrogencarbonate (NH_4_HCO_3_)	Gaseous	WRR: 99.9%.	[65]
2019	Inorganic fertilizers (KCl and NH_4_PO_3_)	Inorganic-based	WRR: >90%.	[66]
2011	Fertilizers (KCl, NaNO_3_, NH_4_Cl, and (NH_4_)_2_SO_4_)	Inorganic-based	——	[54]
2019	Mg(NO_3_)_2_ 6H_2_O	Inorganic-based	93% P recovery; 50% N recovery.	[67]
2017	Iron(III) sulfate	Inorganic-based	WF: 3.75 L/m^2^·h for brackish water; WF: 1.61 L/m^2^·h for seawater.	[64]
2021	Food additives (monosodium glutamate (MSG), saccharin (SAS), and trisodium citrate (TSC))	Organic-based	WF: up to about 20 L/m^2^·h for all the draw solutes.	[68]
2021	Molasses	Organic-based	WF: 16.7 L/m^2^·h for deionized water; WF: 13.3 L/m^2^·h for brackish wastewater; WF: 7.5 L/m^2^·h for seawater.	[69]
2019	Ethanol	Organic-based	——	[70]
2015	Polyacrylamide	Organic-based	——	[71]
2021	d-xylose-coated MNPs	MNP-based	WF: 2.98 L/m^2^·h.	[72]
2021	Fe_3_O_4_ nanoparticles with sodium alginate (SA)	MNP-based	WF: 13.8 L/m^2^·h.	[73]
2015	Electro-responsive polymer hydrogels	Hydrogel	WF: 26.47 L/m^2^·h.	[74]
2011	Ionic polymer hydrogel	Hydrogel	WF: lower than 1 L/m^2^·h.	[75]

WF is water flux; WRR means water recovery rate.

**Table 2:** CA FO membrane fabrication in the last ten years.

Year	Membranes	Materials	Preparation Methods	Membrane Performance	References
2013	PVA-coated flat-sheet CA	PVA; CA	Phase inversion	WF: improved 20% compared with pure CA; SR: not changed.	[94]
2013	CTA/CA	CTA; CA	Immersion precipitation	WF: 10.39 L/m^2^·h, RSF: 0.084 mol NaCl/m^2^·h (FS: Milli-Q water; DS: 1 M NaCl); WF: 9.27 L/m^2^·h, RSF: 0.248 mol KBr /m^2^·h (FS: Milli-Q water; DS: 1 M KBr); lower membrane fouling.	[95]
2015	fCNT-CA	fCNT; CA	Phase inversion	WF: 50% higher than the unmodified CA; advanced alginate fouling resistance.	[58]
2018	CA modified with PVA and PDA	PVA; PDA; CA	Phase inversion	WF: 16.72 L/m^2^·h, RSF: 0.14 mol NaCl/m^2^·h, Js/Jw: 3 times lower than that of bare CA (FS: DI water; DS: 2.0 M NaCl).	[96]
2018	Flat-sheet CA	CA	Phase inversion via immersion precipitation	WF: 21.75 L/m^2^·h, RSF: 5.88 g/m^2^·h; WF: 42.25 L/m^2^·h, RSF: 17.66 g/m^2^·h (Prediction based on Box–Behnken design).	[97]

SR represents salt rejection; RSF denotes reverse solute flux; Js/Jw equals SR to WF; DS means draw solution; FS is feed solution.

**Table 3:** TFC FO membrane fabrication in the last ten years.

Year	Membranes	Materials	Preparation Methods	Membrane Performance	References
2011	TFC	PSf support; PA active layer	Phase separation and interfacial polymerization	WF: 4–25 L/m^2^·h, SR: over 95.5% (DS: 1 M NaCl).	[98]
2011	flat-sheet TFC	Porous PSf substrates, PA rejection layers	Phase inversion and interfacial polymerization	WF: 54 L/m^2^·h, 50% higher than the commercial CTA-HW, (DS: 2 M NaCl).	[90]
2011	TFC modified with PDA	BW30, SW30-XLE; PET fabric layer; Psu	——	WF: a two-fold increase for the SW30-XLE but a reduction for the BW30; an 8–15-fold increase compared to the control data	[99]
2012	Zeolite-PA TFN; Zeolite-PA TFC	PSf substrates; PA active layer; NaY zeolite nanoparticles.	Phase inversion and interfacial polymerization	WF: 80% increase compared to the pure TFC (DS: 0.5 M NaCl;FS: 1 M NaCl).	[100]
2013	TFC supported by nylon 6,6 MF membrane	nylon 6,6 MF membrane support; Poly(piperazinamide) or PA selective layer	Interfacial polymerization	WF: matched, RSF: 10-fold lower than the HTI CA.	[101]
2014	TiO_2_ TFN	PSf matrix; PA rejection layer; TiO_2_ nanoparticles	Phase inversion and interfacial polymerization	WF: improved by 86–93%. (DS: 1 M NaCl;FS: 2 M NaCl).	[102]
2015	PAN hollow-fiber supported TFC	Hydrophilic PAN hollow fiber support; PA active layer.	Dry-jet wet-spinning; interfacial polymerization	WF: 36.6 L/m^2^·h in PRO (DS: 1 M NaCl; FS: DI water).	[103]
2016	Anti-biofouling GO/Ag TFN	TFC; PA active layer; graphite powder; AgNO_3_	——	——	[104]
2017	GO/PA TFN	PSf support; PA active layer; GO nanosheets	Phase inversion and interfacial polymerization	WF: 14.5 L/m^2^·h in FO, RSF: reduced up to 39%.	[36]
2017	PES/PDA UF	PES UF membrane; PA layer; PDA	Phase inversion	WF: 166 L/m^2^·h, SR: BSA rejection of 92.9%, 20.23 mg Pb/g, 17.01 mg Cd/g and 10.42 mg Cu/g.	[88]
2018	TFC	PSf polymer; PA active layer	Phase inversion and interfacial polymerization	SR: phenol rejection of 79% and benzene rejection of 90%.	[105]
2018	GO/MWCNT TFN	Nylon MF substrates; GO; MWCNT	Interfacial polymerization	WF: increase, RSF: reduce.	[106]
2019	HTI CTA, TFC-AQP flat-sheet membranes	——	Direct purchase	SR: boron rejection of 98.4% and iodide rejection of 98.3% (DS: 2 M MgCl_2_).	[107]
2020	GO/PA-TFN	PSf Substrate; PA layer; Graphite	Phase inversion and interfacial polymerization	WF: increased 56.97% in AL-FS mode and 42.48% in AL-DS, SR: chlorine resistance 75 times better than baseline membrane (DS: 2 M NaCl).	[108]
2020	GO/PA-PEG/PSf TFN	PSf substrates; PA layer; GO; PEG.	Phase inversion and interfacial polymerization	WF: 34.3 L/m^2^·h SR: rejection rates of benzene, phenol, and toluene (97, 84, and 91%, respectively).	[109]
2021	GO/PVA hydrogel-coated PA TFC	PA TFC; GO; PVA.	Phase inversion and interfacial polymerization	WF: 29.3 L/m^2^·h; RSF: 13.8 g/m^2^·h; SRSF: 0.46 g/L.	[110]
2022	Fe_3_O_4_/fCNT-embedded PA TFC	polysulfone fibers; PA layer; Fe_3_O_4_ particle; fCNTs.		WF: 27.4 L/m^2^·h; SR: rejections of 90% and 94% for Na_2_SO_4_ and NaCl.	[111]

**Table 4:** Recent PBI membrane fabrication in FO.

Year	Membranes	Materials	Preparation Methods	Membrane Performance	References
2007	PBI NF hollow-fiber membranes	hollow fiber; PBI dope	Dry-jet wet phase inversion and chemically cross-linking modification.	High water permeation flux and high rejection to divalent ions.	[116]
2009	PBI NF hollow-fiber membranes	PBI dope	Dry-jet wet phase inversion and chemically cross-linking modification.	High permeation flux and improved salt selectivity.	[117]
2013	PBI flat-sheet membranes	PBI dope	Dip-coating and phase inversion.	WF: increase, SR: increase, (DS: 2 M NH_4_HCO_3_; FS: 0.1 M NaCl).	[118]
2013	PBI-POSS/PAN	(1) CA; (2) PBI/PES; (3) PBI-POSS/PAN	——	WF: 1.3% flux reduction, minimal scaling.	[119]
2013	PBI/POSS-PAN/PVP dual-layer hollow-fiber membranes	PBI/POSS outer layer; PAN/PVP inner layer	——	WF: 31.37 L/m^2^·h (DS: 2.0 M MgCl_2_); power density: 2.47 W/m^2^ (DS: 1.0 M NaCl).	[120]
2014	PBI–POSS/PAN hollow-fiber membranes	Hollow-fiber membrane; POSS particles; PAN; PBI	——	RSF: high, Lower fouling.	[121]
2016	Cross-linked N-substituted PBI membrane	N-butylsulfonated PBI; divinyl sulfone	Deprotonation and cross-linking modification.	WF: 22.1 L/m^2^·h, SR: 46% NaCl rejection.	[122]
2020	PBI/SiO_2_ flat-sheet membrane	Polyester fabric; PBI dope; silica nanoparticles	Non-solvent induced phase separation method.	WF: 16.9 L/m^2^·h (twofold higher than the pristine PBI (about 7.4 L/m^2^·h)).	[123]

## Data Availability

Not applicable.
